# Classification of neocortical interneurons using affinity propagation

**DOI:** 10.3389/fncir.2013.00185

**Published:** 2013-12-03

**Authors:** Roberto Santana, Laura M. McGarry, Concha Bielza, Pedro Larrañaga, Rafael Yuste

**Affiliations:** ^1^Departamento de Inteligencia Artificial, Facultad de Informática, Universidad Politécnica de MadridMadrid, Spain; ^2^Intelligent Systems Group, Department of Computer Science and Artificial Intelligence, University of The Basque CountrySan Sebastian, Spain; ^3^Department Biological Sciences, Columbia UniversityNew York, NY, USA

**Keywords:** affinity propagation, cortex, interneurons, cell types

## Abstract

In spite of over a century of research on cortical circuits, it is still unknown how many classes of cortical neurons exist. In fact, neuronal classification is a difficult problem because it is unclear how to designate a neuronal cell class and what are the best characteristics to define them. Recently, unsupervised classifications using cluster analysis based on morphological, physiological, or molecular characteristics, have provided quantitative and unbiased identification of distinct neuronal subtypes, when applied to selected datasets. However, better and more robust classification methods are needed for increasingly complex and larger datasets. Here, we explored the use of affinity propagation, a recently developed unsupervised classification algorithm imported from machine learning, which gives a representative example or exemplar for each cluster. As a case study, we applied affinity propagation to a test dataset of 337 interneurons belonging to four subtypes, previously identified based on morphological and physiological characteristics. We found that affinity propagation correctly classified most of the neurons in a blind, non-supervised manner. Affinity propagation outperformed Ward's method, a current standard clustering approach, in classifying the neurons into 4 subtypes. Affinity propagation could therefore be used in future studies to validly classify neurons, as a first step to help reverse engineer neural circuits.

## Introduction

To properly understand the structure and function of any circuit it seems essential to objectively define its elements. Unfortunately, as opposed to elements in electronic circuits, neurons in brain circuits do not come pre-labeled and it is not clear exactly what comprises a neuronal cell type. GABAergic neocortical interneurons are a particularly difficult case, due to their large molecular, morphological and physiological diversity (Fairen et al., 1984; Mott and Dingledine, 2003; Ascoli et al., 2008). In the past, cell type classification was a qualitative and subjective task that led to inconsistent classes of neurons. Recently, quantitative methods using unsupervised cluster analysis have become standard for classification of neurons (Cauli et al., 1997; Karube et al., 2004; Ma et al., 2006; Dumitriu et al., 2007; Helmstaedter et al., 2009; Karagiannis et al., 2009; McGarry et al., 2010; DeFelipe et al., 2013). In particular, traditional cluster analysis using Ward's method has been effective, but it is a simple technique with some drawbacks. Hierarchical agglomerative clustering is a bottom–up technique, that is, it starts by grouping the two “closest” cells as defined by the algorithm, then joins the next “closest” sets and so on. As a hierarchical clustering technique the grouping decisions it makes are inflexible so, once two cells are joined together they remain joined in the final hierarchy. Moreover, hierarchical methods are susceptible to a chaining effect, where cells are sometimes assigned to existing clusters rather than being grouped in new clusters. These qualities of the method could pose limitations as it prevents it from testing multiple possible groupings of the dataset.

A new, and more sophisticated, classification method called affinity propagation does not have those limitations, is an unsupervised algorithm, and has demonstrated success with greatly improved results over standard methods in other classification problems (Frey and Dueck, 2007). In particular, affinity propagation goes beyond the solution of the neuronal classification problem and solves a related but also difficult problem: the identification of exemplars, i.e., elements that best characterize each class (Mézard, 2007). In neuroscience, affinity propagation was recently used for the analysis of spike synchronization in rat brains (Takahashia et al., 2010), in learning the role of sleep slow wave activity in visuomotor learning (Landsness et al., 2009), and in the discovery of synaptic connectivity organizational principles (Perin et al., 2011). When applied to neuronal classifications, affinity propagation could therefore identify exemplar neurons to be used as a compressed, characteristic representation of each neuron subtypes. This seems advantageous, since focusing the analysis on exemplars could ease the burden of analyzing hundreds, or thousands of neurons, for the identification of salient characteristics of neuron classes.

In this work we explored the application of affinity propagation to neuronal classification, by using the algorithm to blindly classify a test dataset of four known interneuron subtypes. The test dataset was comprised of 67 morphological and 20 electrophysiological variables (Supplementary Table 1) describing (1) parvalbumin-positive (PV+) basket cells (BC), (2) PV+ chandelier cells (ChC), (3) somatostatin-positive (SOM+) Martinotti cells (MC), and (4) SOM+ non-Martinotti cells (non-MC), as described in (McGarry et al., 2010). We found that affinity propagation generates an accurate classification in separating these four known interneuron subtypes. Our data suggest that affinity propagation could be a powerful new classification tool for discovering or defining neuronal cell types.

## Materials, methods, and protocol

### Preparation of brain slices

Acute brain slices were prepared from Nkx2.1, G42, or GIN mice, with an average age of 15 postnatal days (range P13–25). Mice were quickly decapitated, the brain was removed and then immediately placed in cold sucrose cutting solution (222 mM sucrose, 2.6 mM KCl, 27 mM NaHCO_3_, 1.5 mM NaH_2_PO_4_, 0.5 mM CaCl_2_, 3 mM MgSO_4_, bubbled with 95% 0_2_, 5%CO_2_). Coronal slices of 300 μm thickness were cut using a Vibratome and transferred to a holding chamber at room temperature with oxygenated ACSF (126 mM NaCl, 3 mM KCl, 3 mM MgSO_4_, 1 mM CaCl_2_, 1.1 mM NaH_2_PO_4_, 26 mM NaHCO_3_, and 10 mM dextrose, bubbled with 95% 0_2_, 5%CO_2_). After at least an additional 30 min equilibration at room temperature, slices were transferred to a recording chamber with perfusion of ACSF bubbled with 95% 0_2_, 5%CO_2_.

### Transgenic mouse lines

To identify different types of interneurons we used three transgenic mouse lines. First, we used the G42 line that labels PV+ cells (Chattopadhyaya et al., 2007). PV+ cells are fast spiking interneurons with basket or ChC morphology. We could identify BC from ChC by their distinctive morphologies and threshold spiking responses. Additionally, to find ChCs we used Nkx2.1 Cre MADM mice (referred to as Nkx2.1 mice) where we could often identify ChC by their distinct axonal arbors, visible with illumination of the GFP (Woodruff et al., 2009). The Nkx2.1 line labels a population of interneurons that express the transcription factor Nkx2.1, labeling interneurons that migrate from the medial ganglionic eminence (MGE), thus including ChCs (Xu et al., 2008). In both G42 and Nkx 2.1 lines, we had a high success rate of finding ChC at the top of layer 2, close to the layer 1 border, where the normally rare ChC were found among GFP+ cells with a probability of 50–70% (Woodruff et al., 2009). Finally, we used the GIN line to label SOM+ cells (Oliva et al., 2000). SOM+ cells are regular spiking interneurons with varied morphology. We previously identified three subtypes of SOM+ interneurons in GIN mice based on morphology and physiology-MC and two novel subtypes (McGarry et al., 2010). Following that work, here we distinguish between the MC and two novel subtypes (non-MC).

### Electrophysiology recordings

Slices were placed in a recording chamber at room temperature with flowing oxygenated ACSF. Pipettes of 3–7 MΩ resistance were pulled from borosilicate glass. Whole cell recordings were taken in current clamp mode. Only cells with healthy resting membrane potential (between −55 and −80 mV) were selected for recording. Supplementary Figure 2 shows a representative set of the traces of the complete dataset.

### Electrophysiology analysis

Twenty variables were measured for each neuron by analysis of the recordings in MATLAB (Supplementary Table 1). Variables describing firing and passive properties were based on the Petilla terminology (Ascoli et al., 2008).

### Histological procedure

Neurons were filled with biocytin by a patch pipette. Slices were kept overnight in 4% paraformaldehyde in 0.1 M phosphate buffer (PB) at 4°C. Slices were then rinsed three times for 5 min per rinse on a shaker in 0.1 M PB. They were placed in 30% sucrose mixture (30 g sucrose dissolved in 50 ml ddH_2_0 and 50 ml 0.24 M PB per 100 ml) for 2 h and then frozen on dry ice in tissue freezing medium. The slices were kept overnight in a −80°C freezer. The slices were defrosted and the tissue freezing medium was removed by three 20 min rinses in 0.1 M PB. Slices were kept in 1% hydrogen peroxide in 0.1 M PB for 30 min to pretreat the tissue, then were rinsed twice in 0.02 M potassium phosphate saline (KPBS) for 20 min. The slices were then kept overnight in Avidin-Biotin-Peroxidase Complex. Next the slices were rinsed three times in 0.02 M KPBS for 20 min each. Each slice was then placed in DAB (0.7 mg/ml 3,3”-diaminobenzidine, 0.2 mg/ml urea hydrogen peroxide, 0.06 M Tris buffer in 0.02 M KPBS) until the slice turned light brown, then immediately transferred to 0.02 M KPBS and transferred again to fresh 0.02 M KPBS after a few minutes. The stained slices were rinsed a final time in 0.02 M KPBS for 20 min. Each slice was observed under a light microscope and then mounted onto a slide using crystal mount.

### Reconstruction and analysis of morphology

Successfully filled and properly stained neurons were then reconstructed using Neurolucida software (MicroBrightField) (Supplementary Figure 1). The neurons were viewed with a 100× oil objective on an Olympus IX71 inverted light microscope or an Olympus BX51 upright light microscope. The neuron's processes were traced manually while the program recorded the coordinates of the tracing to create a digital three-dimensional reconstruction. In addition to the neuron, the pia and white matter were drawn. The Neurolucida Explorer program was used to measure 67 morphological variables of the reconstruction describing somatic, dendritic, and axonal properties. (Supplementary Table 1).

### Affinity propagation

Affinity propagation is a clustering algorithm that, given a set of points and a set of similarity values between the points, finds clusters of similar points, and for each cluster gives a representative example called an exemplar (Frey and Dueck, 2007). Affinity propagation has several advantages over related techniques. Methods such as *k*-centers clustering and *k*-means clustering store a relatively small set of estimated cluster centers at each step. These techniques can be improved by using methods that begin with a large number of clusters and then prune them, but they still rely on random sampling and make hard pruning decisions that cannot be recovered from. In contrast, by simultaneously considering all data points as candidate centers and gradually identifying clusters, affinity propagation is able to avoid many of the poor solutions caused by unlucky initializations and hard decisions (Frey and Dueck, 2007).

A characteristic that makes affinity propagation different from other clustering algorithms is that the points directly exchange information between them regarding the suitability of each point to serve as an exemplar for a subset of other points. The algorithm takes as input a matrix of similarity measures between each pair of points *s*(*i, k*). Instead of requiring that the number of clusters be predetermined, affinity propagation takes as input a real number *s*(*k, k*) for each data point *k*. These values, which are called preferences, are a measure of how likely each point is to be chosen as exemplar. In our case the preference can be understood as a particular weight given to each neuron according to a priori knowledge of the suitability of the neurons to be exemplars. This parameter can be used to bias the clustering procedure when there are some neurons that are known to be good descriptors. However, in our experiments we did not assume any a priori information and all neurons where given the same preference. The algorithm works by exchanging messages between the points until a stop condition, which reflects an agreement between all the points with respect to the current assignment of the exemplars, is satisfied. These messages can be seen as the way the points share local information in the gradual determination of the exemplars.

There are two types of messages to be exchanged between data points. The responsibility *r*(*i, k*), sent from data point *i* to candidate exemplar point *k*, reflects the accumulated evidence for how well-suited point *k* is to serve as the exemplar for point *i*, taking into account other potential exemplars for point *i*. The availability *a*(*i, k*), sent from candidate exemplar point *k* to point *i*, reflects the accumulated evidence for how appropriate it would be for point *i* to choose point *k* as its exemplar, taking into account the support from other points that point *k* should be an exemplar.

The availabilities are initialized to zero: *a*(*i,k*) = 0. Then, the responsibilities are computed using the rule:

(1) $$r(i,k)\leftarrow s(i,k)-\max_{k^{\prime }|k^{\prime }\neq k}\left\{a(i,k^{\prime })+s(i,k^{\prime })\right\}$$

The responsibility update shown in Equation (1) allows all the candidate exemplars compete for ownership of a data point. Evidence about whether each candidate exemplar would make a good exemplar is obtained from the application of the following availability update:

(2) $$a(i,k)\leftarrow \min\left\{0,r(k,k)+\sum_{i^{\prime }\neq i,k}\max\left\{0,r(i^{\prime },k)\right\}\right\}$$

In the availability update shown in Equation (2) only the positive portions of incoming responsibilities are added, because it is only necessary for a good exemplar to explain some data points (positive responsibilities), regardless of how poorly it explains points with negative responsibilities. To limit the influence of incoming positive responsibilities, the total sum is thresholded so that it cannot go above zero.

The self-availability *a*(*k, k*) is updated differently:

(3) $$a(k,k)\leftarrow \sum_{i^{\prime }\neq k}\max\left\{0,r(i^{\prime },k)\right\}$$

For a point *i*, the value of *k* that maximizes *a*(*i, k*) + *r*(*i, k*) either identifies point i as an exemplar if *k* = i, or identifies the data point that is the exemplar for point *i*.

Update rules described by Equations (1), (2), and (3) require only local computations. The message-passing procedure may be terminated after a fixed number of iterations, when changes in the messages fall below a threshold, or after the local decisions stay constant for some number of iterations.

Similarly to other propagation methods, damping should be used to confront numerical oscillations that arise in some circumstances. This technique consists of setting each message to λ times its value from the previous iteration plus 1 − λ times its prescribed updated value (0 < λ < 1). A pseudocode of our approach for neuron classification using the affinity propagation algorithm is shown in **Algorithm 1**.

**Table:** 

Algorithm 1: Neuron classification using affinity propagation
1. Normalize each of the neuron features to values between 0 and 1.
2. Find the similarity values between pairs of neurons using a predefined distance.
3. Compute the preference values for each neuron.
4. Cluster neurons using affinity propagation.
5. Assign to all neurons the class determined by its exemplar.
6. Compute the classification accuracy.

To compute the similarity measure (step 2 in Algorithm 1), the Spearman distance, i.e., one minus the sample Spearman's rank correlation between observations (treated as sequences of values), was used.

The similarity measure is computed as the opposite of the distance, *s*(*i, k*) = –*d*(*i, k*). Original settings of the affinity propagation algorithm are used (Frey and Dueck, 2007). The preference values (step 3 of Algorithm 1) for all points *s*(*k, k*) is computed as the median value of the similarity values *s*(*i, j*).

To measure the agreement between a clustering produced by affinity propagation and a priori known set of labels for the points, a convention has to be set. We decide that the exemplar of each cluster will determine the putative label of all points in the cluster. Consequently, the classification accuracy (step 6 of Algorithm 1) is computed as the proportion of points whose putative label agrees with its true class.

We include a number of remarks concerning the application of **Algorithm 1**:

- The output of affinity propagation (step 4 of Algorithm 1) depends on its input parameters. In particular it is sensitive to the similarity values between the points and the preference of each point to become a cluster. This means that changing these input parameters may determine changes in the number of clusters and their composition.
- To evaluate the quality of a clustering produced by affinity propagation, two aspects should be simultaneously considered: (1) the number of points that are correctly classified. Since we assume that the labels of the exemplars are known, the correctly classified points may artificially increase. Therefore, we compute the classification accuracy as the ratio between correctly classified points (excluding the exemplars) and the total number of points (excluding the exemplars); and (2) the number of clusters, which should be preferably few.

## Results

### Database of four known interneuron subtypes

We explored the use of affinity propagation to classify neocortical GABAergic neurons based on their morphological and physiological properties. In order to test the affinity propagation algorithm we used a well-characterized database where the group identity of the neurons was known from previous studies (McGarry et al., 2010; Packer and Yuste, 2011; Woodruff et al., 2011). Specifically, we used a physiology database which contained 337 interneurons neurons distributed as: 63 BC, 218 ChC, 40 MC, and 16 somatostatin positive interneurons non-MC. The morphology database contained 109 neurons distributed as: 31 BC, 23 ChC, 33 MC, and 22 non-MC. Finally, there were 50 neurons in a joint Morphology+Physiology database, formed by intersecting both databases. Its distribution was: 11 BC, 23 ChC, 9 MC, and 7 non-MC.

### Affinity propagation classification of interneuron morphologies

**Figure 1:**
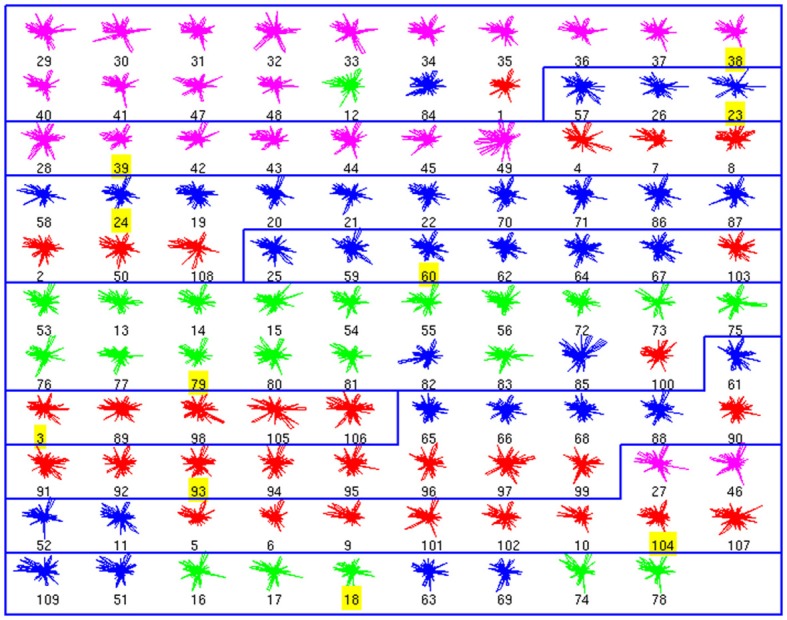
**Morphological Clusters**. Neurons are represented as colored glyphs. Colors red, blue, green, and magenta, respectively, represent neuron types BC, MC, non-MC, and ChC. The label of the exemplar in each cluster is shaded in yellow. Ten clusters are found; most clusters are dominated by a neuron type. BC and ChC (red and magenta) are closely related PV+ interneurons and MC and non-MC (blue and green) are both subtypes of SOM+ cells. Note how cluster 3 (from top) groups BC and ChC jointly and cluster 10 (last one) groups MC and non-MC together.

Using these databases as ground truth, we performed affinity propagation, blind to the cell types. We analyzed the clusters structure, starting with the morphological database (Figure 1). In these figures, each neuron is shown as a colored glyph, i.e., a star plot that represents each neuron as a “star” whose *i*th spoke is proportional in length to its *i*th feature value. Similar shaped glyphs correspond to neurons with similar values of their features. The colors provide additional information about the a priori known neuron class, i.e., red is BC, magenta is ChC, blue is MC and green is non-MC. Good clusters are those where neurons in the same cluster share the same color. BC and ChC (red and magenta) are closely related PV+ interneurons and MC and non-MC (blue and green) are both subtypes of SOM+ cells.

The analysis of the morphology database resulted in 10 clusters (Figure 1). For example, the first cluster included 17 neurons, with 14 ChCs, had three misclassified neurons, those with numbers 12 (of non-MC type, in green), 84 (MC, in blue), and 1 (BC, in red). Its representative or exemplar was neuron 38 (a ChC). Clusters 3, 4, 5, 6, 7, 9, and 10 were also mixed, although in most of them, a single cell type also dominated. Clusters 2 and 8 did not contain any error. To measure the accuracy of the classification, we counted the correctly classified neurons in each cluster. Overall, 73 neurons out of 99 are correctly classified (once the exemplars are not considered), yielding an accuracy of 0.73 (Table 1).

**Table 1:** **Accuracies computed for the Morphology, Physiology, and Morphology + Physiology databases**.

**Morphology database**		**Physiology database**		**Morphology+Physiology database**	
**ncluster**	**ACC**	**ncluster**	**ACC**	**ncluster**	**ACC**
10	0.7374	36	0.8505	8	0.7857

*ncluster is the number of clusters found by the algorithm. ACC, Accuracy obtained using the four known classes of neurons (BC, ChC, MC, non-MC)*.

### Affinity propagation classification of interneuron physiologies

**Figure 2:**
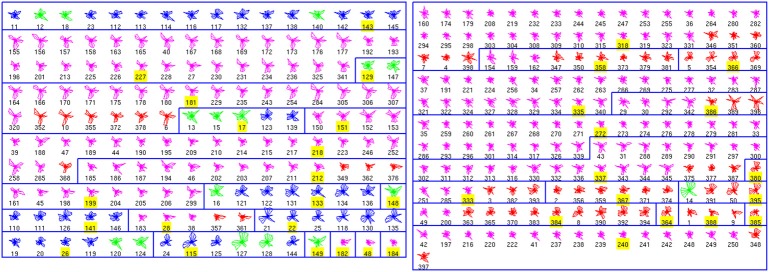
**Physiological Clusters**. Code as in Figure 1. See text for details on the clusters.

We performed a similar analysis of the physiological database, finding 36 clusters (Figure 2). Seven clusters (numbers 11, 17, 18, 19, 20, 28, 33, and 35) are only composed by one cell. In contrast, other clusters include many neurons. Moreover, some of these crowded clusters contained ChC neurons (magenta; clusters 2, 9, 24, and 26), suggesting a potential method of identifying new subgroups of ChC cells, by analyzing some representative electrophysiological features (see below). Overall, the accuracy in this database was 0.85 (255 correctly classified out of 301; again with exemplars not considered in this calculation).

### Affinity propagation classification of interneuron joint databases

**Figure 3:**
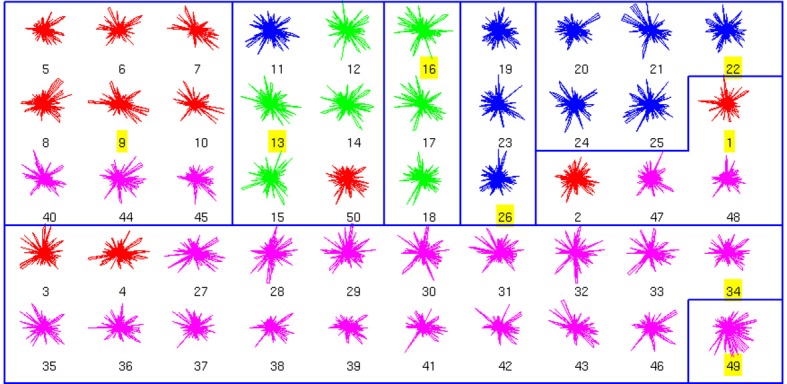
**Clusters of the combined Morphology+Physiology database**. Code as in Figure 1. See text for details on the clusters.

We also performed an analysis of the combined anatomical and physiological databases, which had fewer neurons (50; Figure 3). We found 8 clusters, dominated by a single cell type. In fact, clusters 3, 4, 5, and 8 had no errors. From a total of 42 non-exemplar neurons, 33 are correctly classified (accuracy of 0.78). Remarkably, the simplest binary distinction parvalbumin/somatostatin distinction was correctly identified in these clusters, with only 1 error out of 50 (neuron number 50, of BC type, in red).

### Defining characteristics of the 4 known interneuron subtypes

As a final step, to extract further information from the clusters produced by the affinity propagation algorithm we identified those features that can serve to characterize each morphological and physiological cluster. The Wilcoxon rank sum test for equal medians was applied to compute features that have a significantly different distribution within each cluster with respect to the distribution in the whole database.

Using this approach, a host of different variables were identified, covering a wide spectrum of morphological and physiological features (Tables 2–4). These features could be useful in providing a compact characterization of those neurons included in the cluster.

**Table 2:**
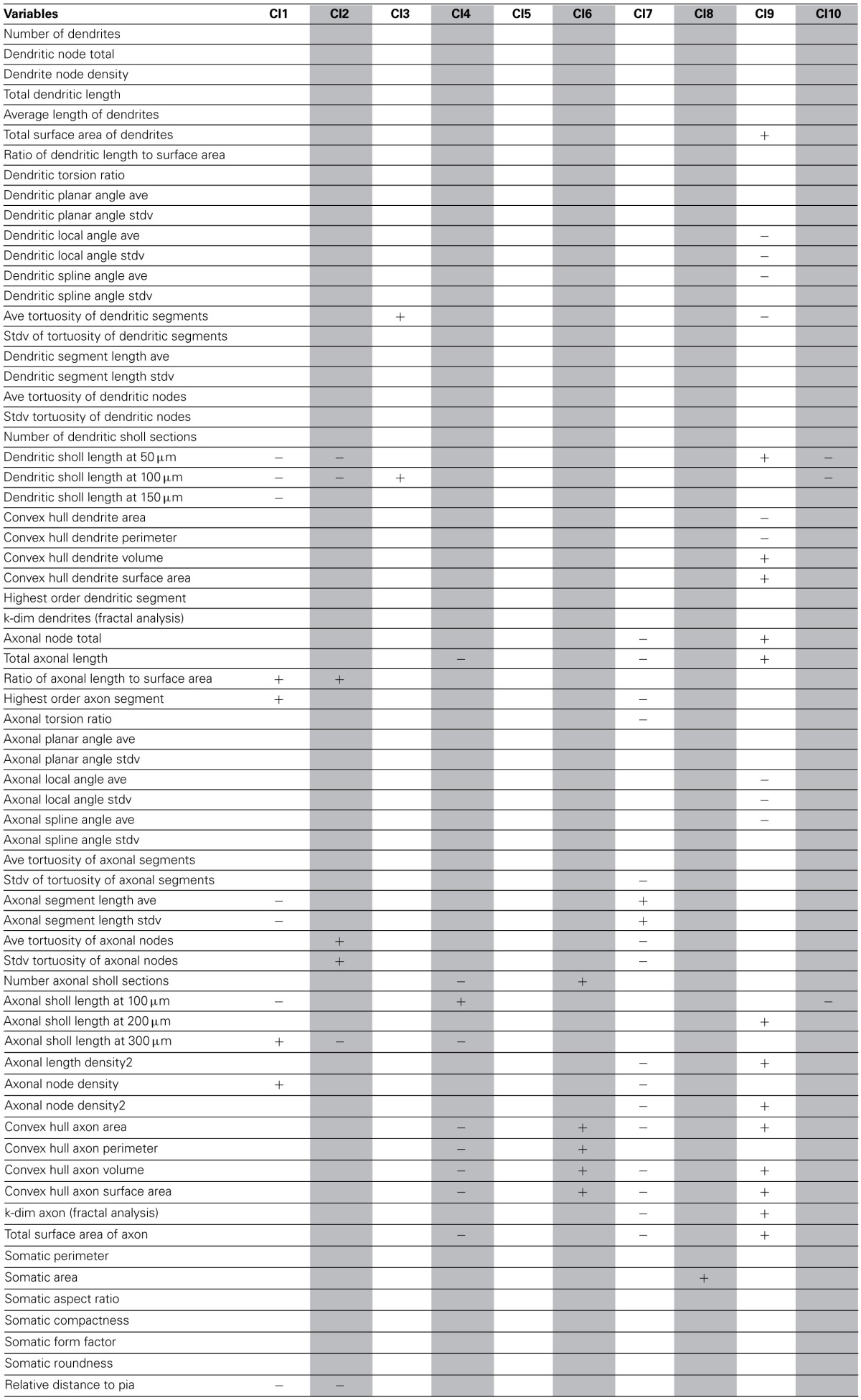
**Significant variables for each morphological cluster**.

*The “+” or “−” signs mean that the average value of a variable in a cluster is significantly higher (or lower), than the average value of that variable in the database*.

**Table 3:**
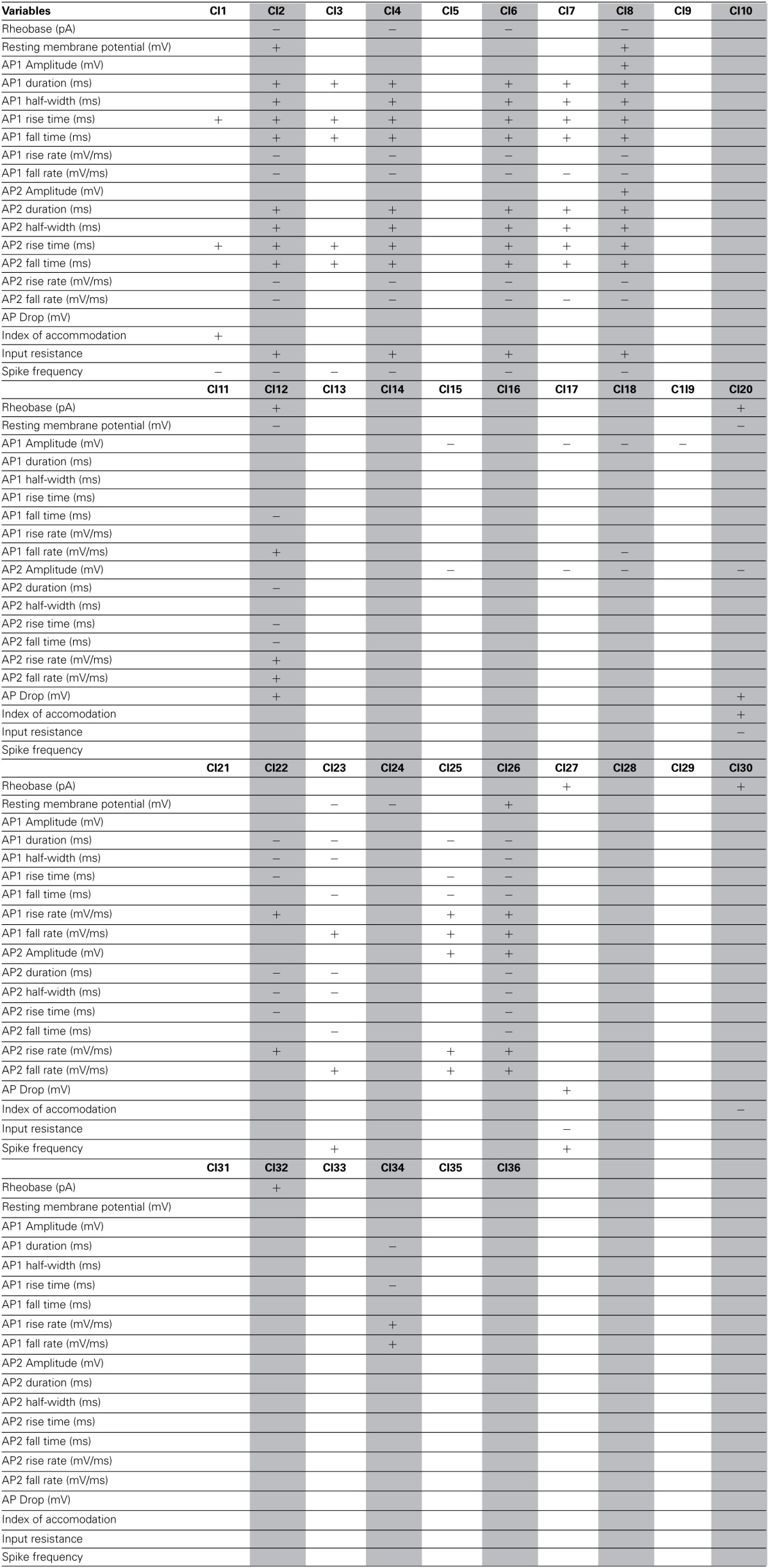
**Significant variables for each physiological cluster**.

**Table 4:**
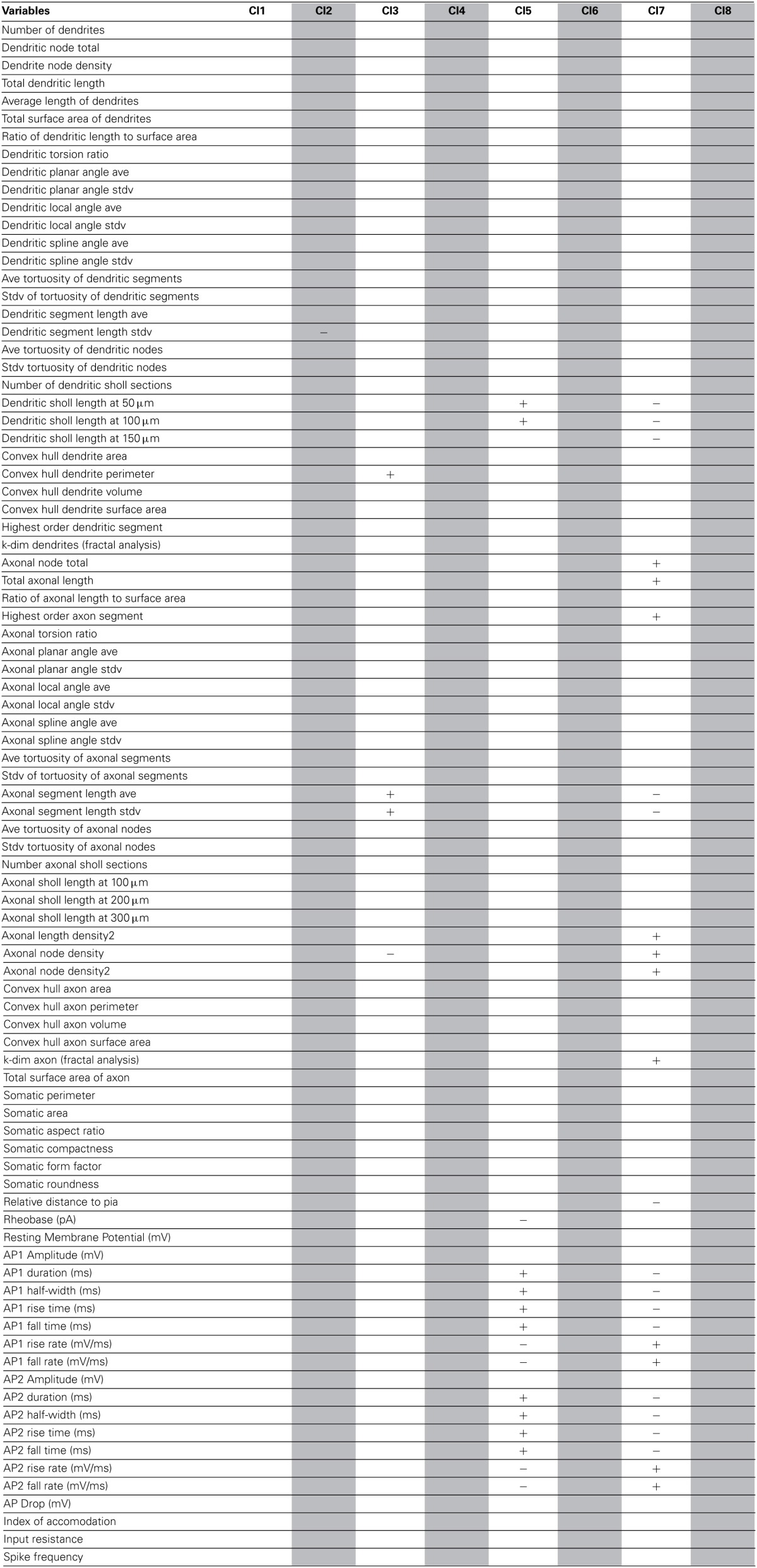
**Significant variables for each cluster of the combined Morphology + Physiology database**.

### Comparison of affinity propagation and ward's method

Ward's method of hierarchical cluster analysis is the current standard used for classifying neuronal cell types. We tested whether affinity propagation improves accuracy over Ward's method. We performed the comparison at three different cluster numbers to illustrate the differences in performance. (1) 4 clusters: To evaluate to what extent 4 clusters found by Ward's method correspond to the four known classes of neurons (PV-basket, PV-chandelier cell, SOM-Martinotti, SOM-non Martinotti). (2) The number of clusters found by affinity propagation (differs for each dataset): To evaluate the accuracy of Ward's method at the same number of clusters as affinity propagation. (3) 2 clusters: To evaluate to what extent Ward's method can separate neurons according to the 2 largest neuron classes (PV vs. SOM).

Accuracy was computed with the respect to the 4 class distinctions for scenarios 1 and 2 and with respect to the 2 class distinction for all three scenarios. The exemplar for Ward's method is computed as the member of the cluster with the smallest mean distance to other members in its cluster. In every case the computation of accuracy was done without counting the exemplar as was done for affinity propagation.

**Figure 4:**
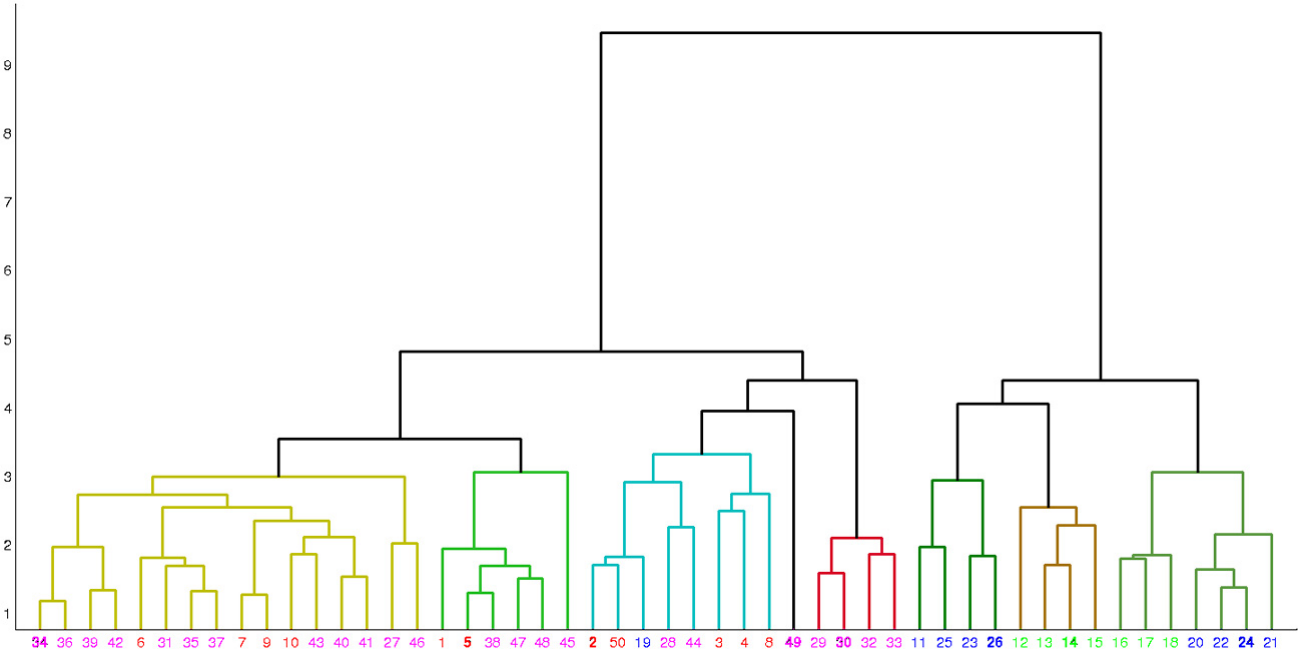
**Hierarchical clustering found by Ward's method for the morphology and physiology database**. Neurons are represented by their index in the database. Colors red, blue, green, and magenta, respectively represent neuron types BC, MC, non-MC and ChC. Neurons are grouped into eight clusters and in each cluster the exemplar is emphasized in bold.

Table 5 shows the comparison of the two classification methods, evaluated for 4 classes (scenarios 1 and 2). Figure 4 shows the hierarchical clustering found by Ward's method for the Morphology+Physiology database. Table 6 shows comparison of the two classification methods evaluated for 2 classes (scenarios 1, 2, and 3).

**Table 5:** **Affinity propagation vs. Ward's method performance**.

**Morphology**		**Physiology**		**Morphology and Physiology**	
**N clusters**	**Accuracy**	**N clusters**	**Accuracy**	**N clusters**	**Accuracy**
**AFFINITY PROPAGATION**					
10	0.7374	36	0.8505	8	0.7857
**WARD'S METHOD**					
4	0.5714	4	0.7575	4	0.6304
10	0.5859	36	0.8510	8	0.6667

*N clusters is the number of clusters. Accuracy is calculated with respect to 4 classes of neurons (BC, ChC, MC, non-MC)*.

**Table 6:** **Affinity propagation vs. Ward's method performance**.

**Morphology**		**Physiology**		**Morphology and Physiology**	
**N clusters**	**Accuracy**	**N clusters**	**Accuracy**	**N clusters**	**Accuracy**
**AFFINITY PROPAGATION**					
10	0.8585	36	0.8471	8	0.9762
**WARD'S METHOD**					
2	0.8037	2	0.9881	2	0.9792
4	0.8000	4	0.9880	4	0.9783
10	0.7879	36	0.9967	8	0.9762

N clusters is the number of clusters. Accuracy is calculated with respect to 2 classes of neurons (PV, SOM)

From these two tables we can see that affinity propagation performs better than Ward's method when accuracy is evaluated on 4 classes, while the performance when accuracy is evaluated on 2 classes is mostly similar between the two methods. Thus, affinity propagation can improve classification for finer distinctions in a dataset. This may be related to the capacity of affinity propagation to identify good descriptors of a subgroup of neurons represented by the exemplars and its ability to pass information between points in the dataset.

## Discussion

### Affinity propagation: a new classification method for neuronal data

In this methodological study we have explored the use of a new algorithm, affinity propagation, for the problem of quantitatively classifying neuronal cell groups. We used a database of 337 neocortical GABAergic interneurons, previously known to belong to four subtypes as “ground truth,” and applied affinity propagation to a collection of morphological and physiological variables measured for each neuron. The classification accuracy we found is overall high: 0.73 for the Morphology database, 0.85 for the Physiology database and 0.78 for the combined Morphology+Physiology database. The accuracy was higher when using physiological information from the neurons, but it contains a larger number of neurons than the other databases, so one would expect that the clustering algorithm will require a higher number of clusters to capture the diversity in the database. Moreover, grouping cells according to a binary parvalbumin/somatostatin class resulted in a much higher accuracy: 85 out of 99 neurons were correctly classified in the morphological database (accuracy of 0.86), and 255 out of 301 in the physiological database (accuracy of 0.85). In this respect, the performance of the affinity propagation algorithm in the combined Morphology+Physiology database was essentially perfect: 49 out of 50 neurons were correctly classified as being parvalbumin or SOM+.

We conclude that affinity propagation can achieve a relatively high accuracy in the classification of interneurons, and is particularly accurate in distinguishing parvalbumin from somatostatin cells. It performs with higher accuracy than Ward's method in further distinguishing the four subtypes of parvalbumin and somatostatin interneurons. This unsupervised method, which already results in high classification accuracies, could improve if combined with dimensionality reduction, as we have found with other unsupervised or supervised neuronal classifications (Guerra et al., 2011).

The extent to which it is useful to subdivide neuronal classes remains a debated subject. Some researchers view neuronal diversity as a continuum of features and refute the existence of discrete subtypes. However, affinity propagation can still be a useful method whether one is a “lumper” or “splitter” of neurons. Affinity propagation does not require an assumption that there are discrete subtypes for clustering. The clusters are produced using exclusively information about the features, not the number of classes. We use the assumption of discrete numbers of neuron types to evaluate the output of the algorithms, but the results—relations between neurons—can be interpreted without this assumption. Additionally even if one takes the view of a continuum of neurons, the exemplars produced by affinity propagation are still valuable since they identify particular neurons that condense information about variations along the continuum.

The identification of exemplars as a part of the clustering algorithm represents a major advantage of affinity propagation. Affinity propagation chooses an exemplar neuron for each cluster that can be studied as a representative of other neurons. The conventional construction of an exemplar for other clustering methods is to compute the centroid of each cluster, computed as having the cluster average for each feature. Clearly these constructed neurons might not be realistic, while the affinity propagation exemplar is a real neuron from the dataset.

While it is possible to pick a real neuron in each cluster as the exemplar as we did for our Ward's method analysis by choosing the neuron with the lowest mean distance to all other neurons in its cluster, affinity propagation has a more robust and informative computation of the exemplar. By including the search for the exemplar as part of the clustering process affinity propagation does not restrict the set of potential candidate exemplars. When calculating exemplars after the clustering is complete, for Ward's method or any other method, the search for candidate exemplars and thus the candidate exemplars of members in that cluster, is restricted to the cluster. Affinity propagation takes into account the whole data set.

### Conflict of interest statement

The authors declare that the research was conducted in the absence of any commercial or financial relationships that could be construed as a potential conflict of interest.

## Supplementary material

Supplementary Figure 1 **Morphological reconstructions of the neurons used**. Ordered according to cell type (ChC, BC, MC, and non-MC).

Supplementary Figure 2 **Electrophysiological recordings of the neurons used**. Ordered according to cell type (ChC, BC, MC, and non-MC).

Supplementary Table 1 **Database of all morphological and electrophysiological variables**.
