# Supplementary figures and images for: Classification of neocortical interneurons using affinity propagation
Source: Front Neural Circuits. 2013 Dec 3;7:185. doi: 10.3389/fncir.2013.00185 (PMC3847556; doi:10.3389/fncir.2013.00185)

115

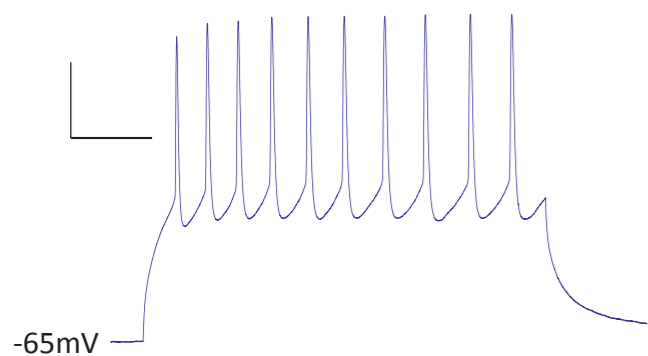

11

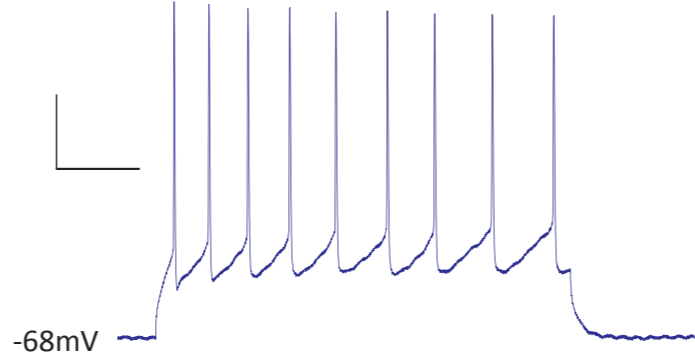

119

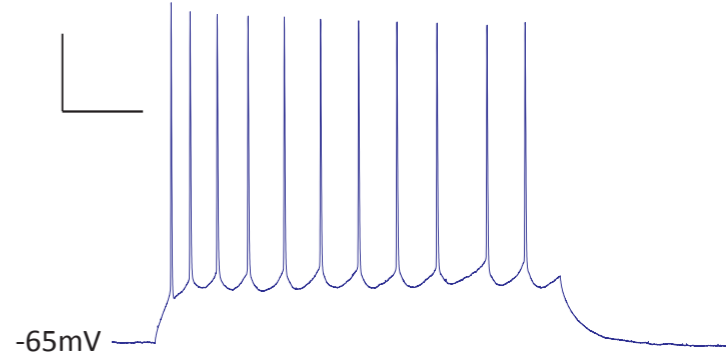

116

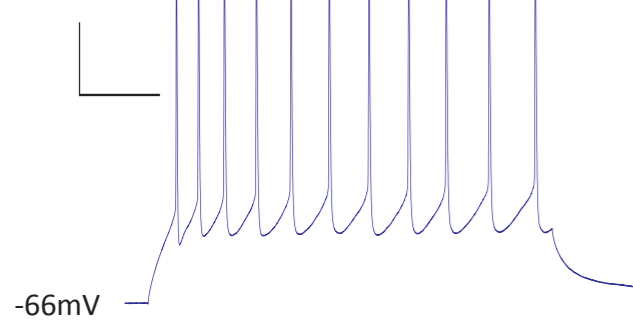

19

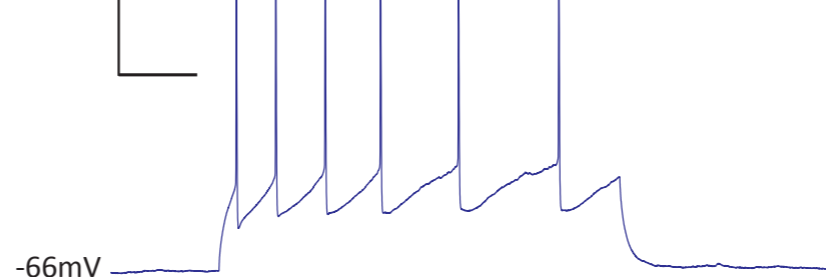

113

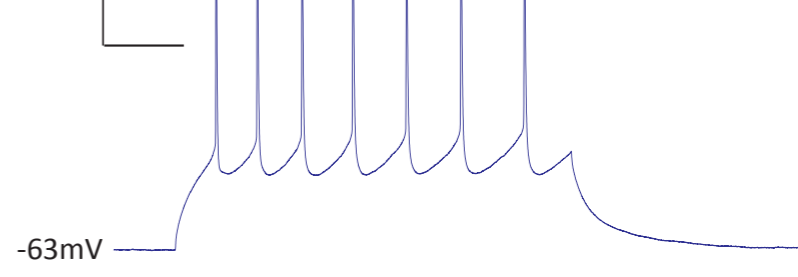

114

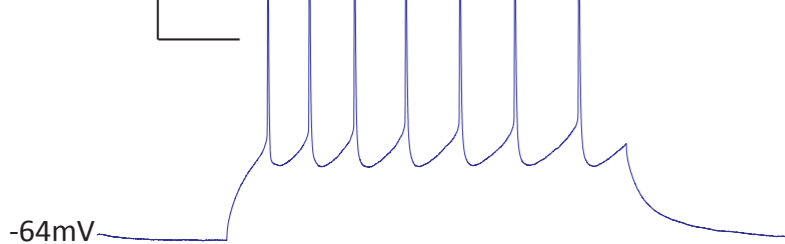

26

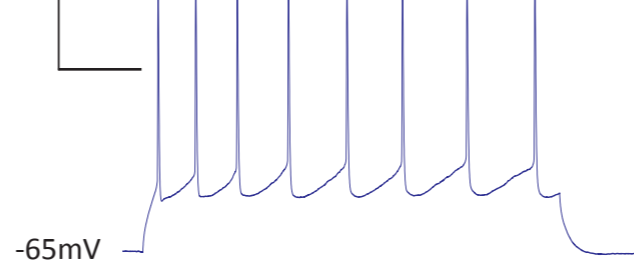

23

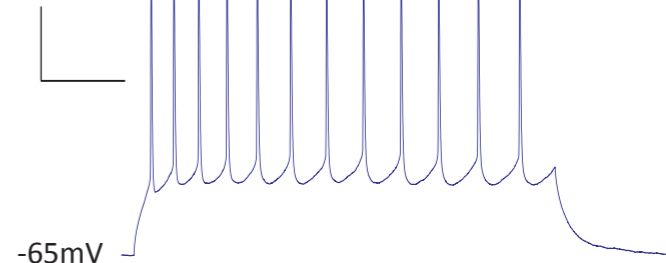

112

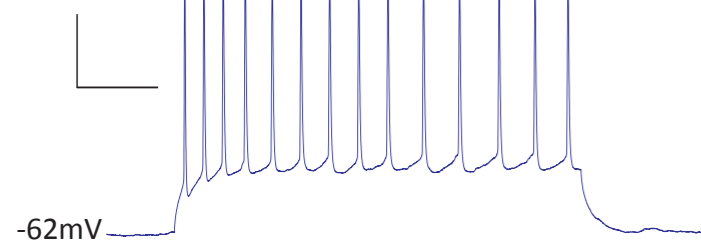

20

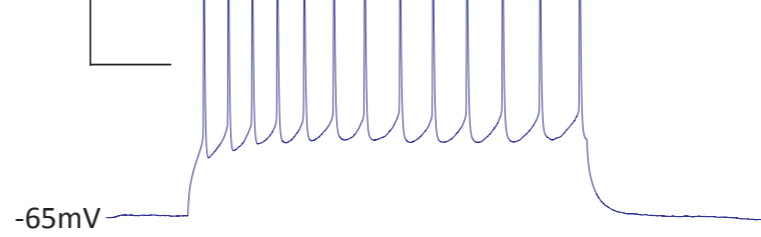

118

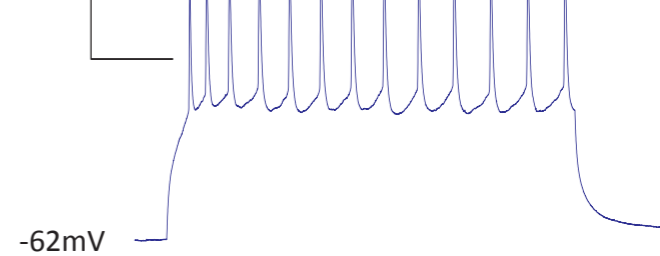

25

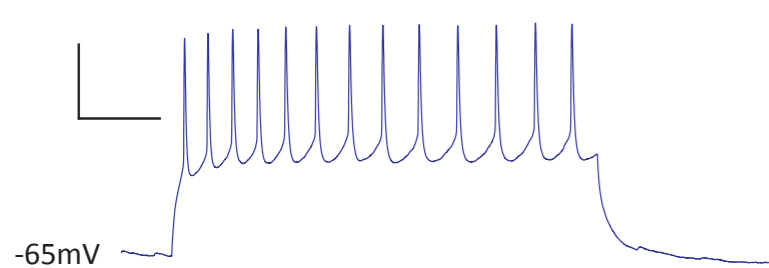

22

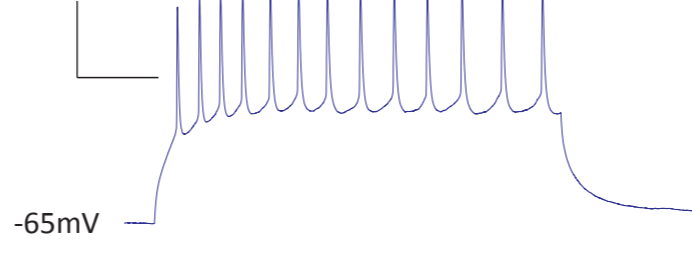

24

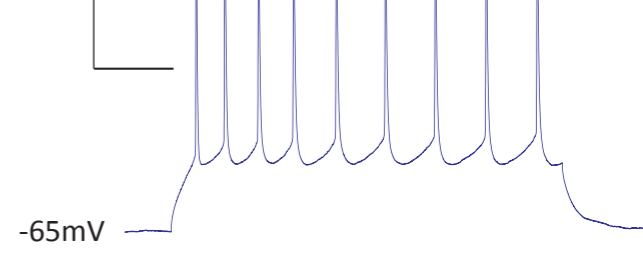

125

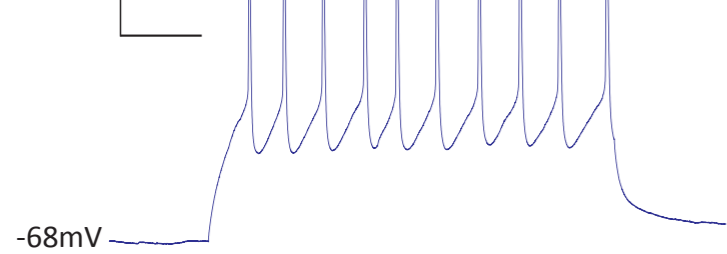

117

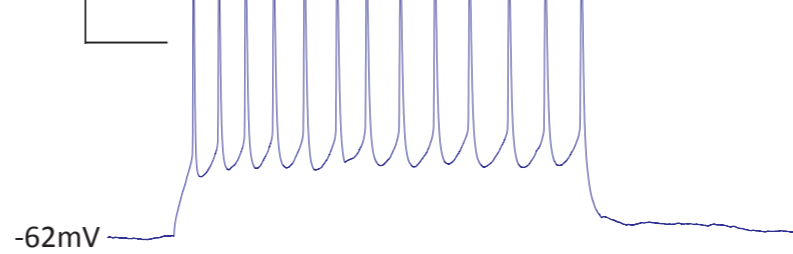

110

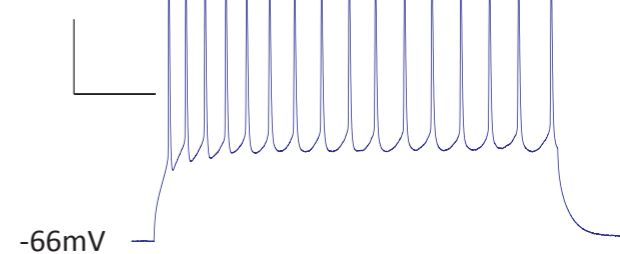

12

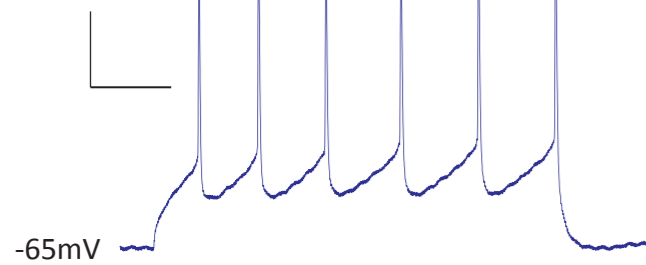

111

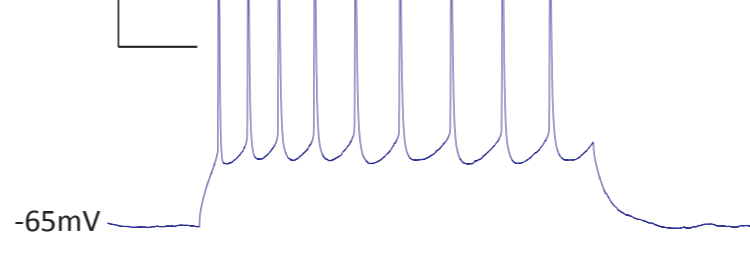

122

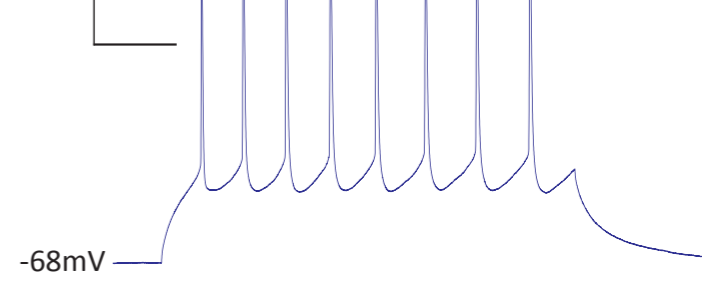

123

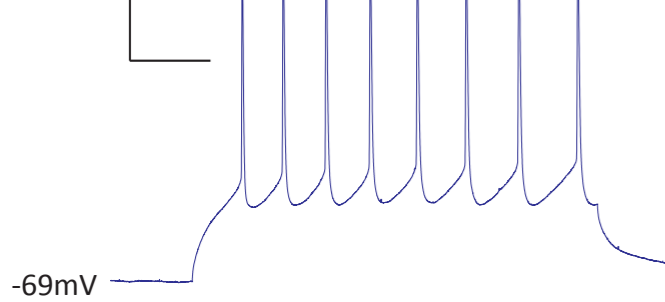

126

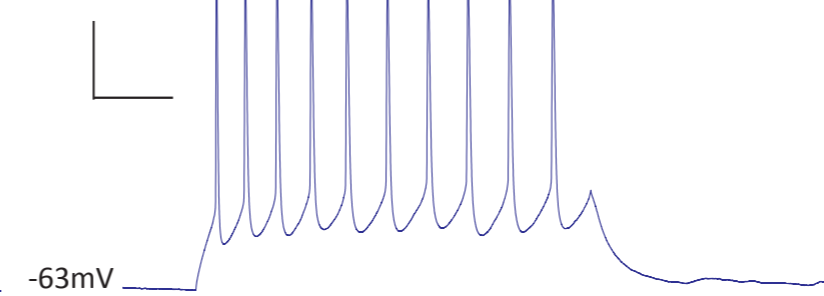

121

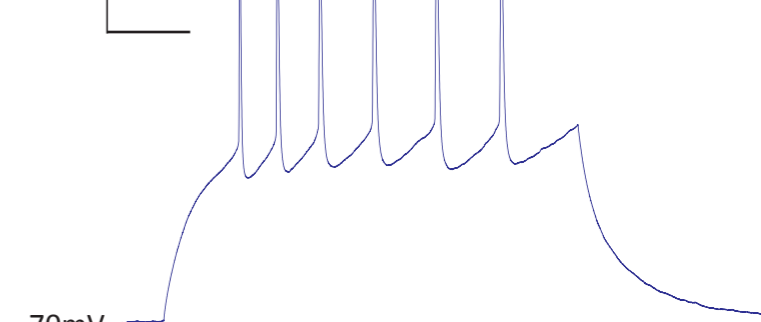

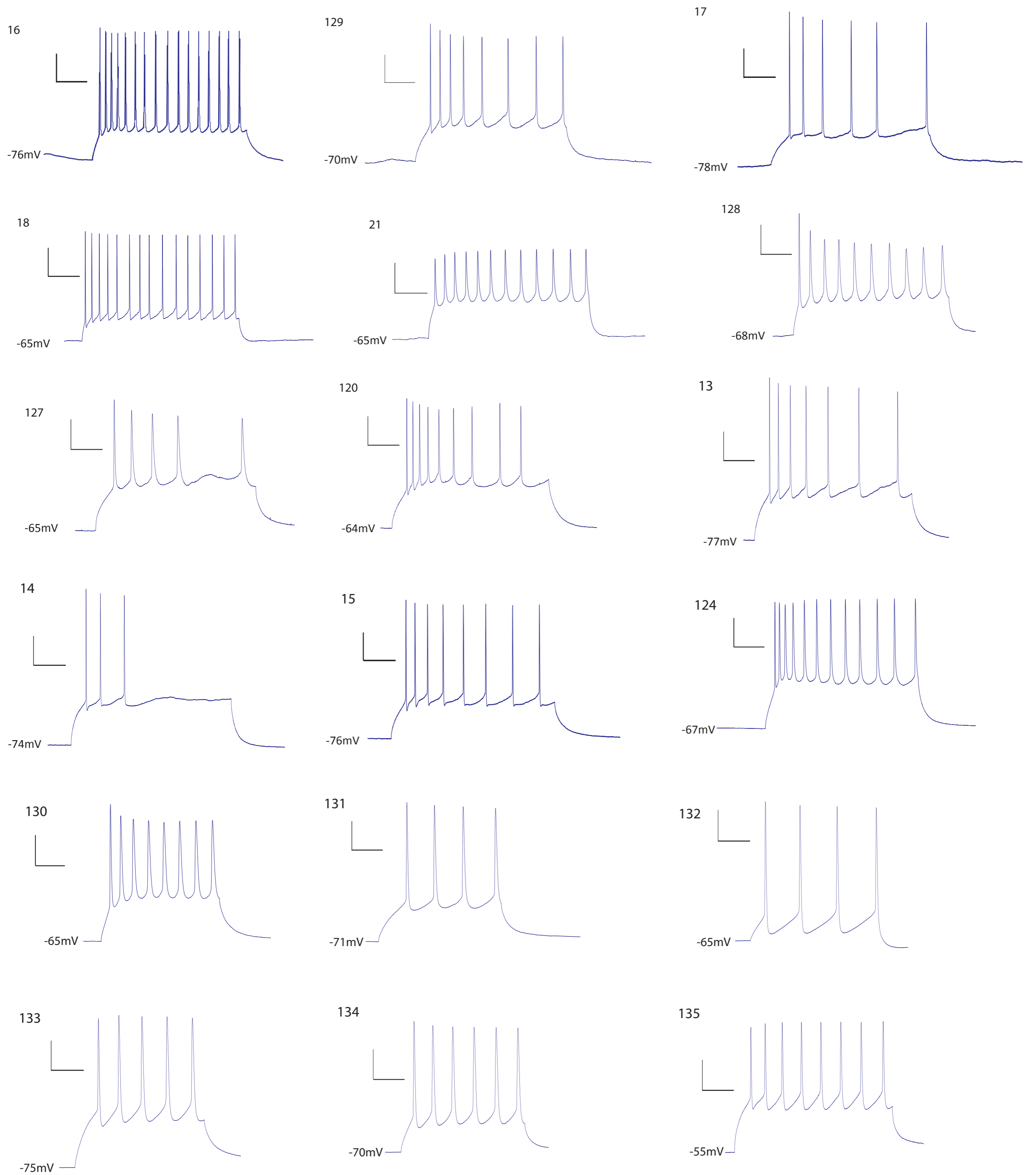

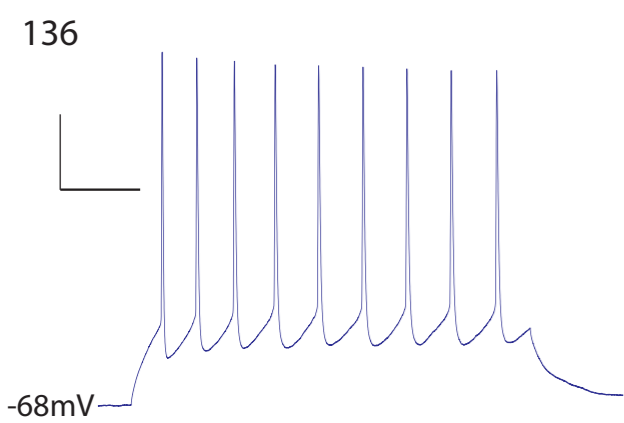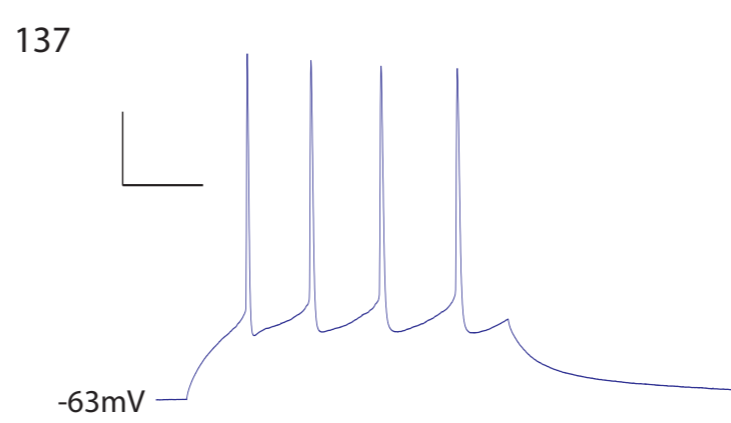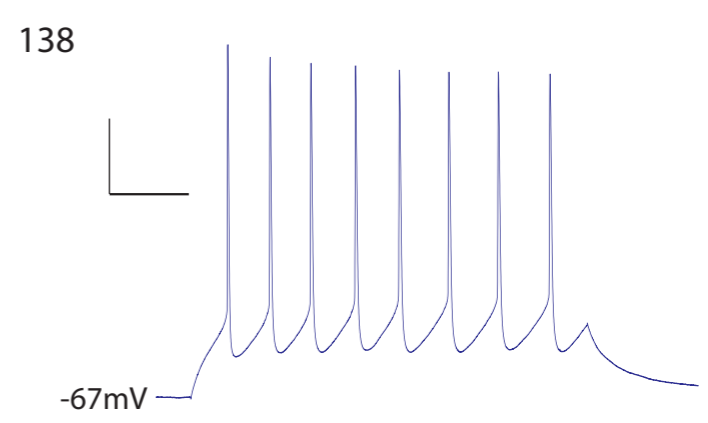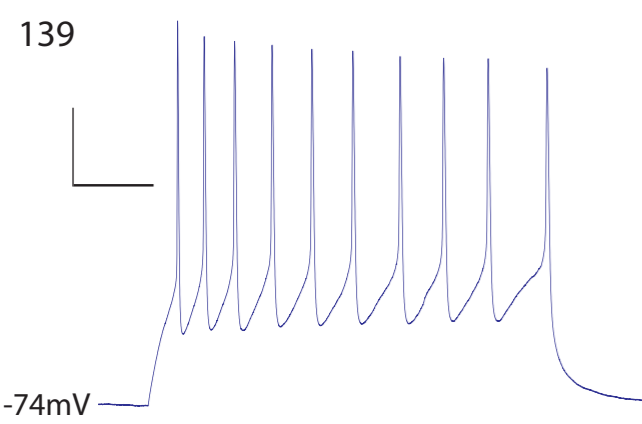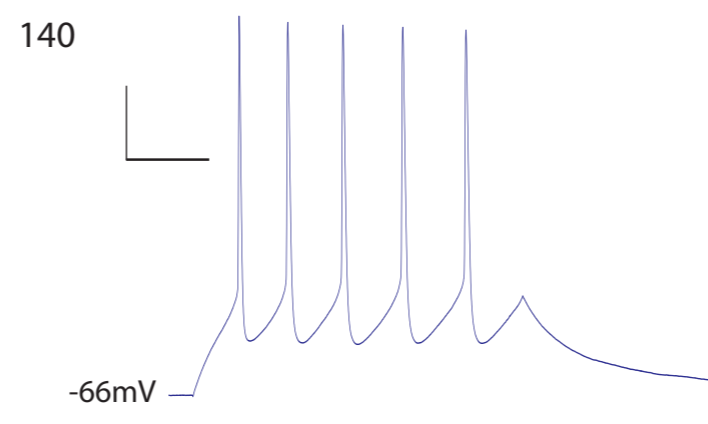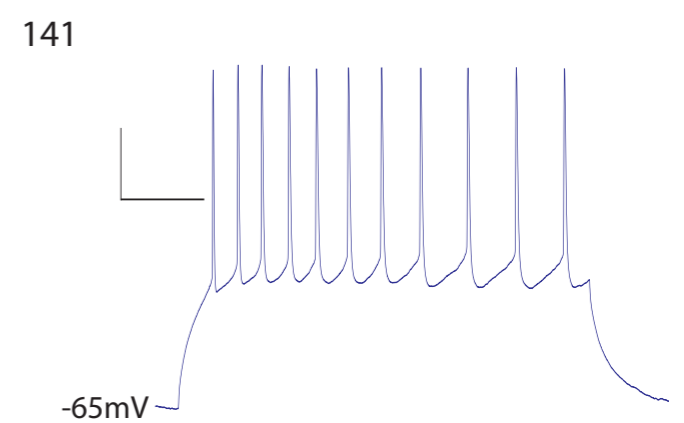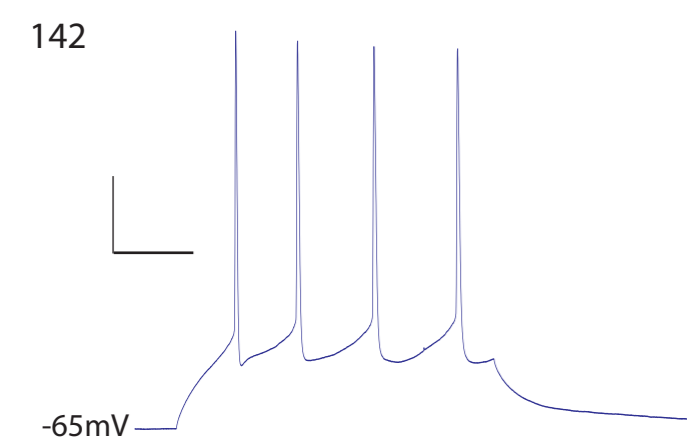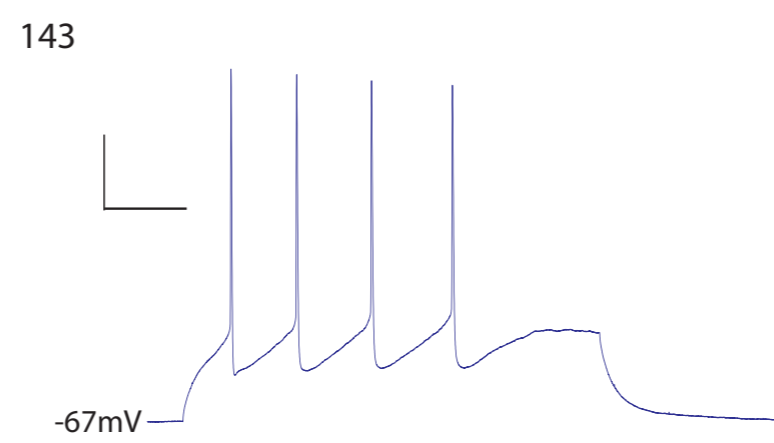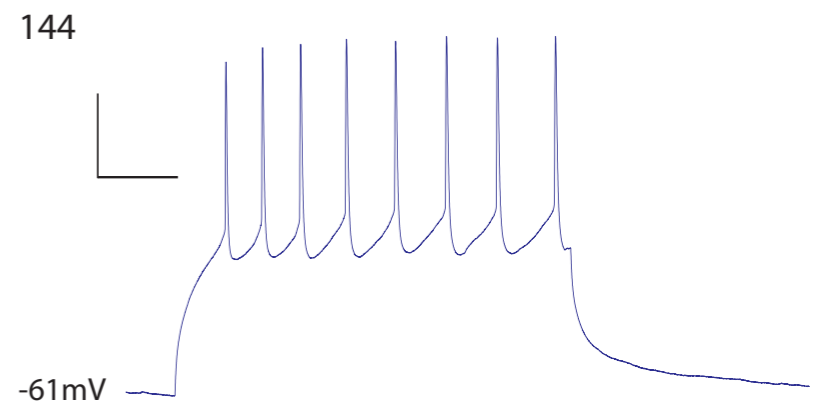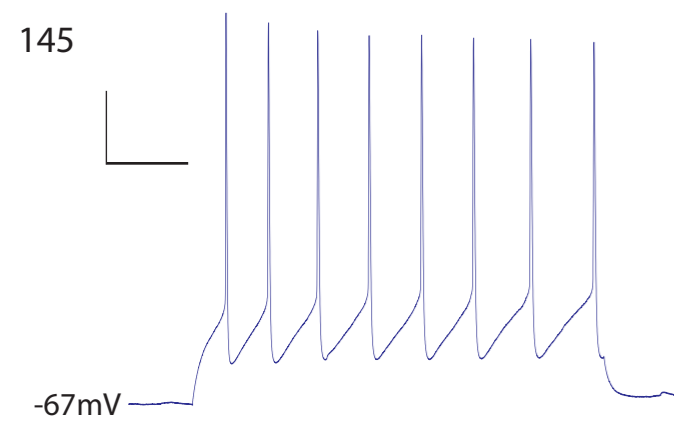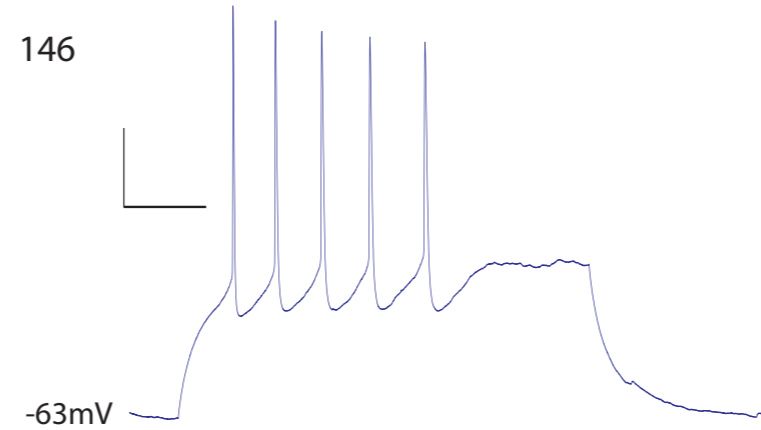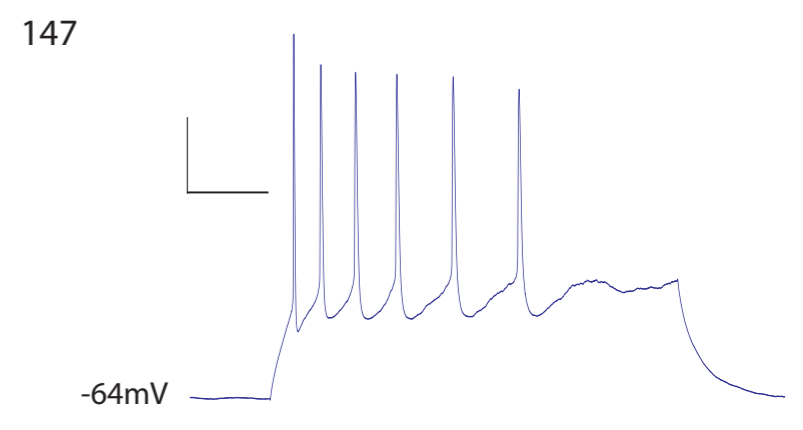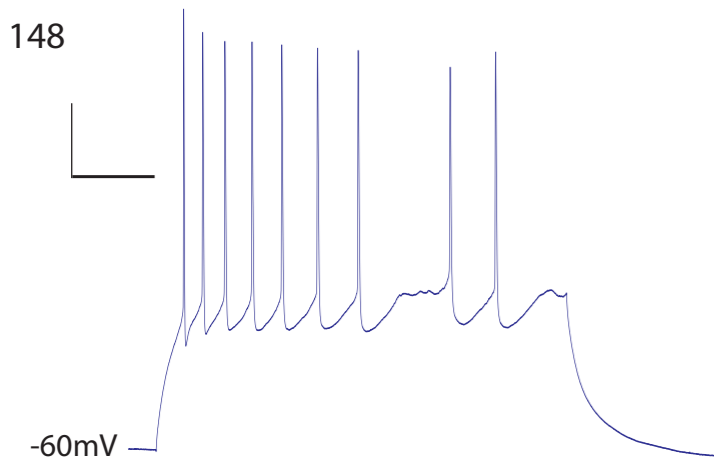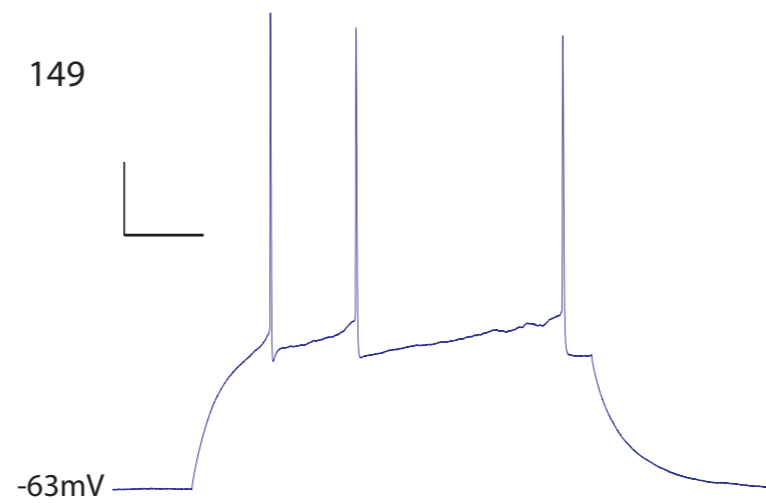

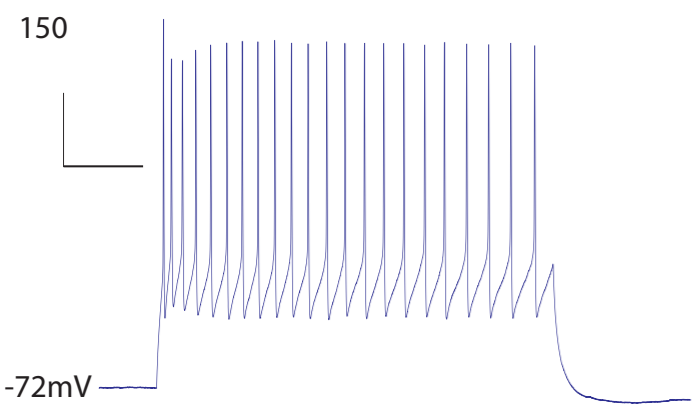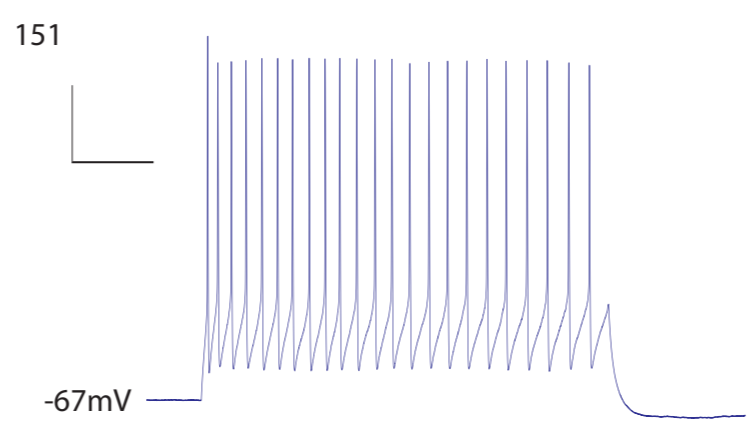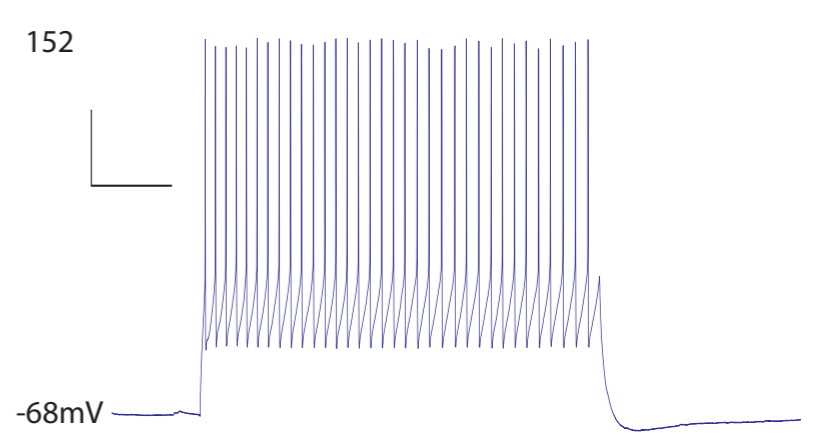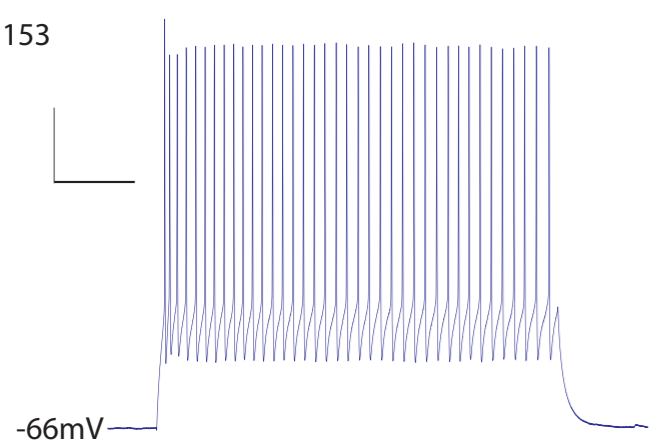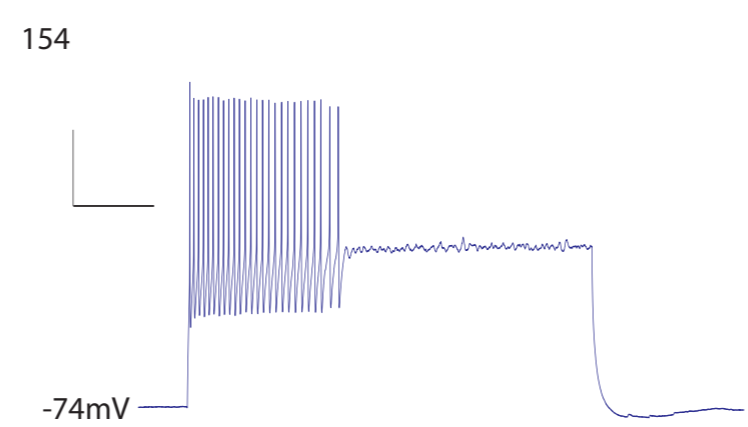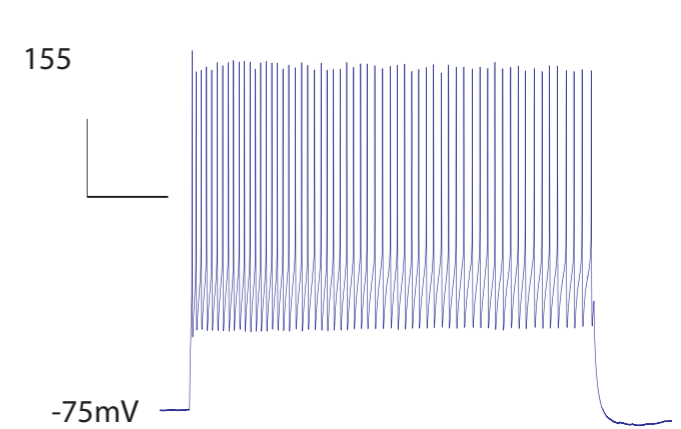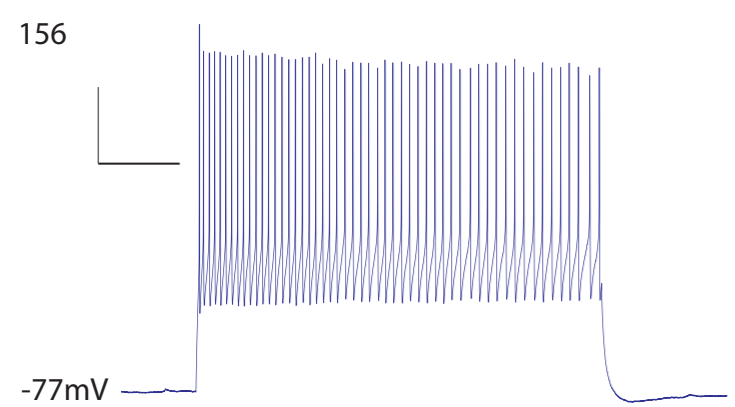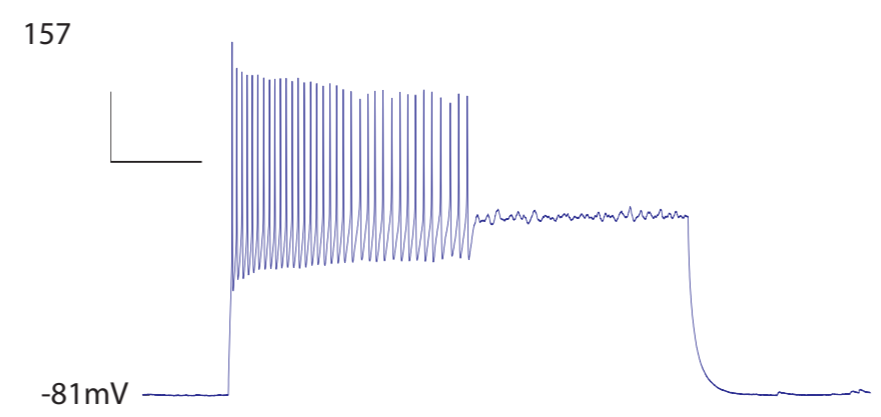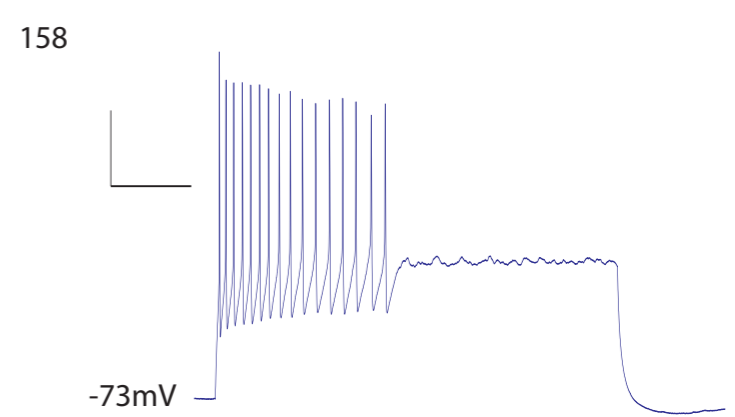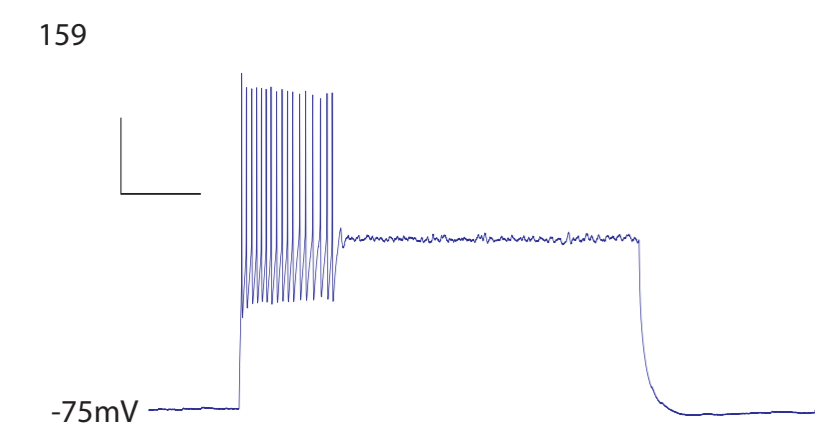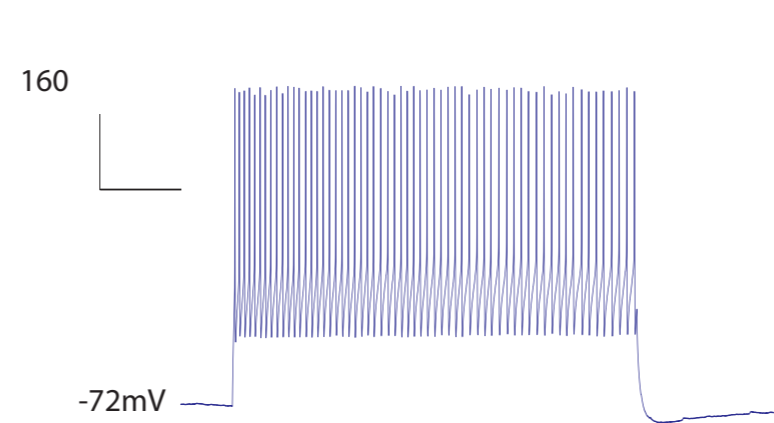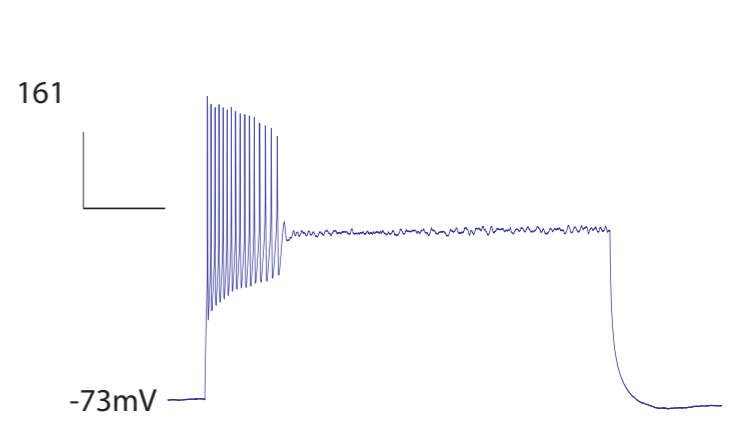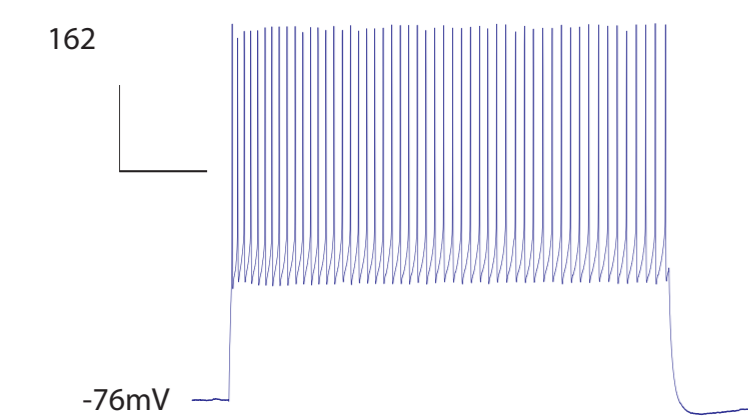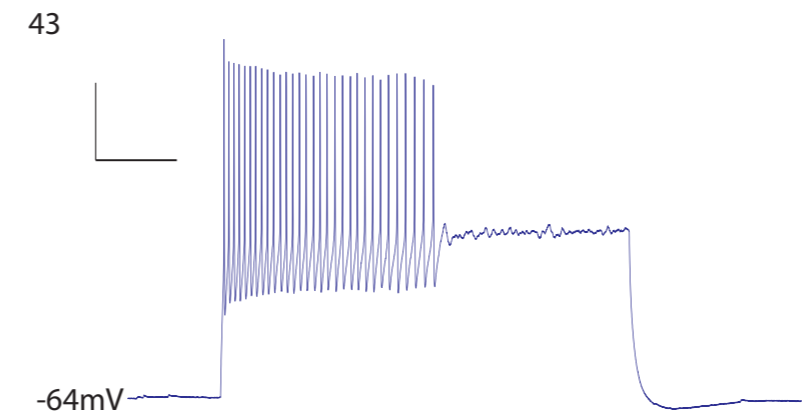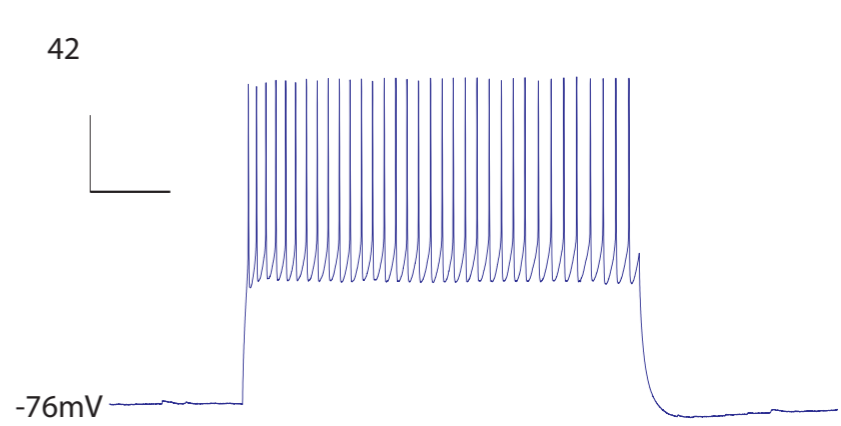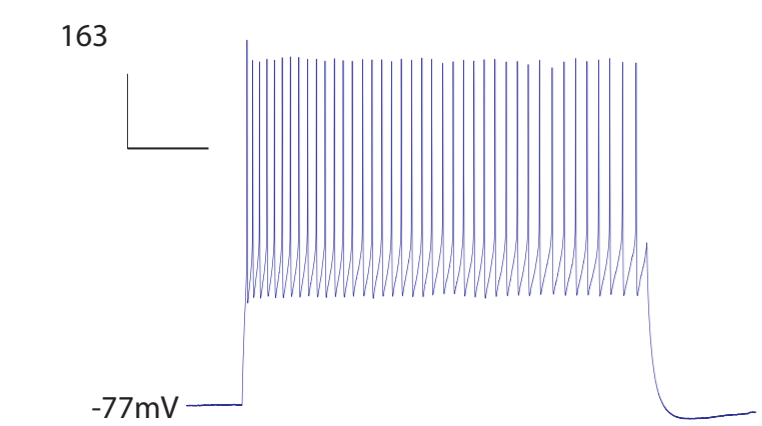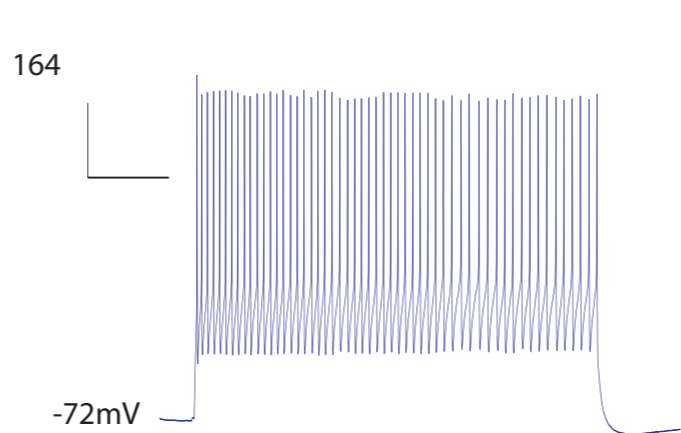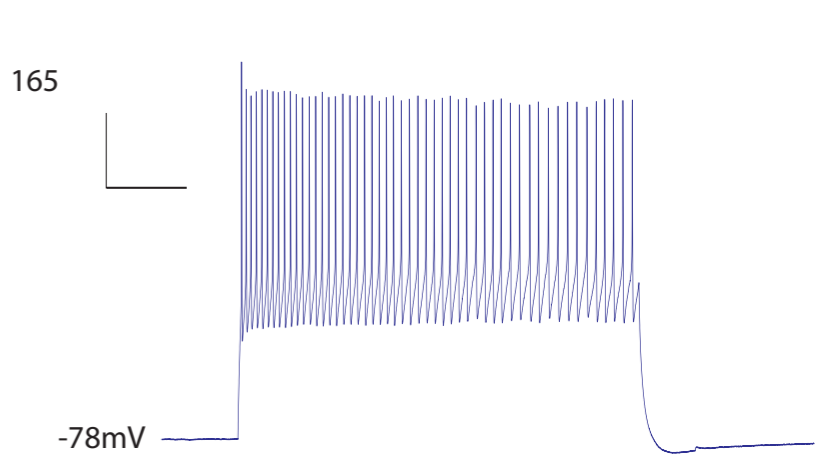

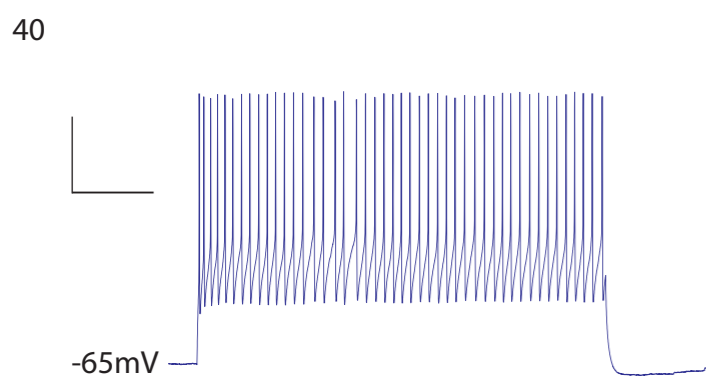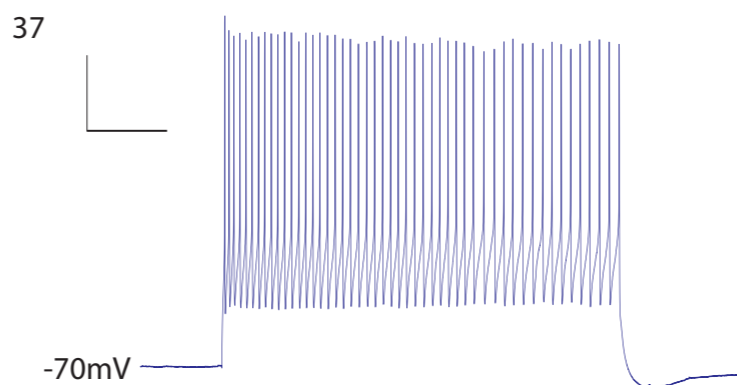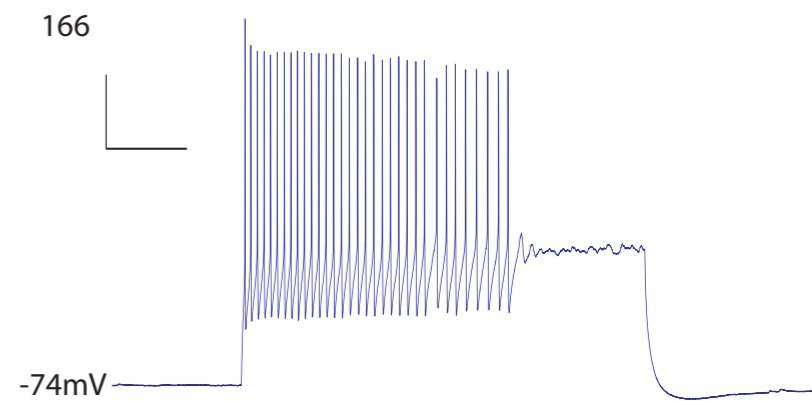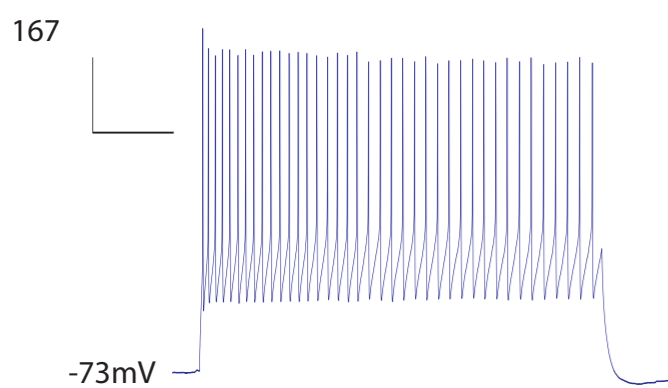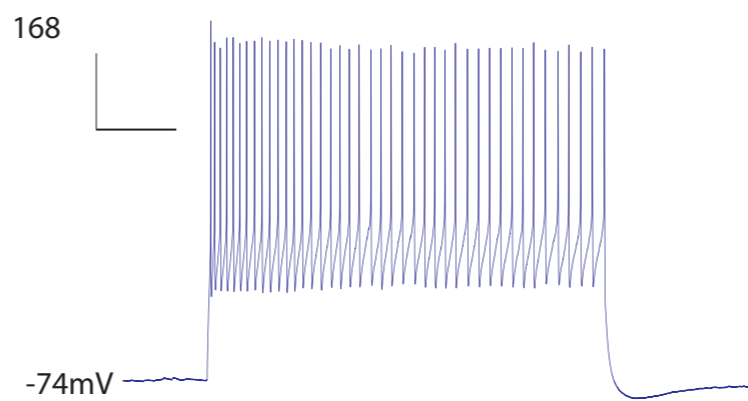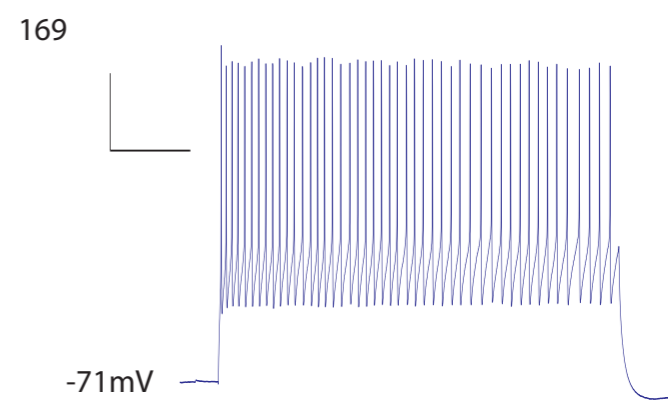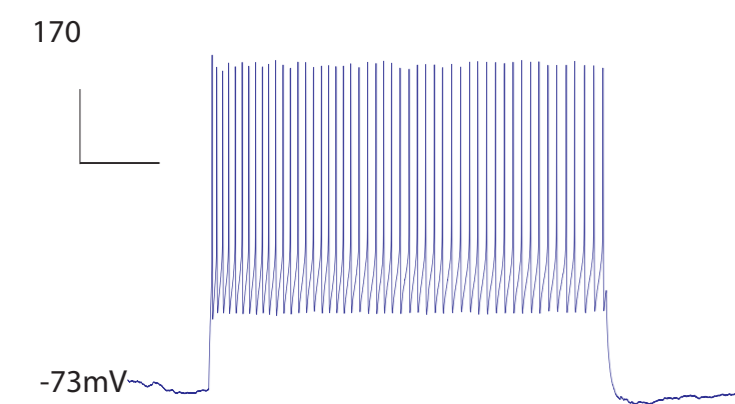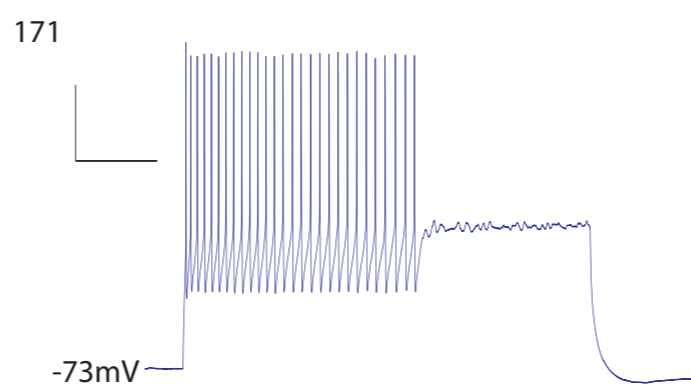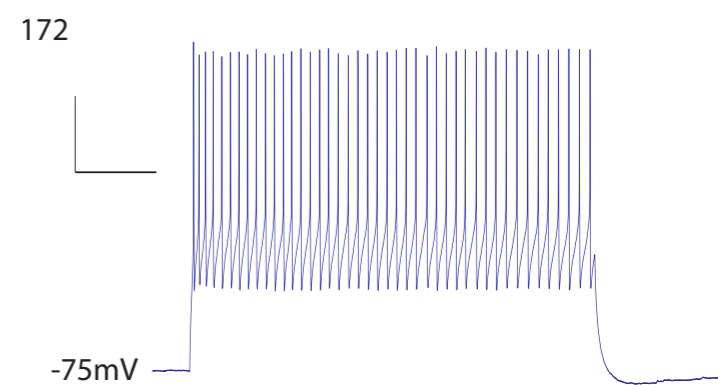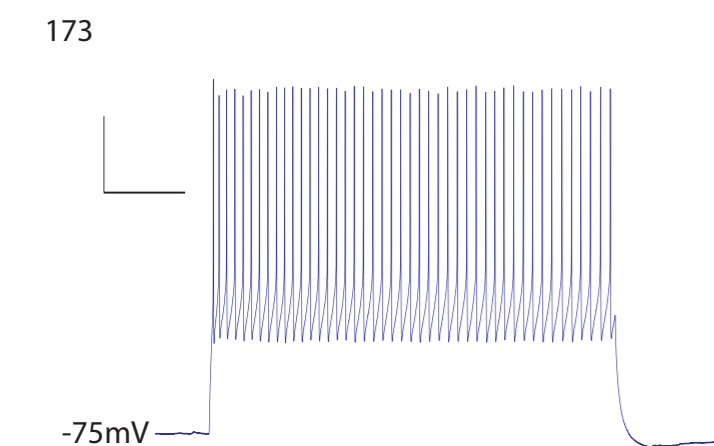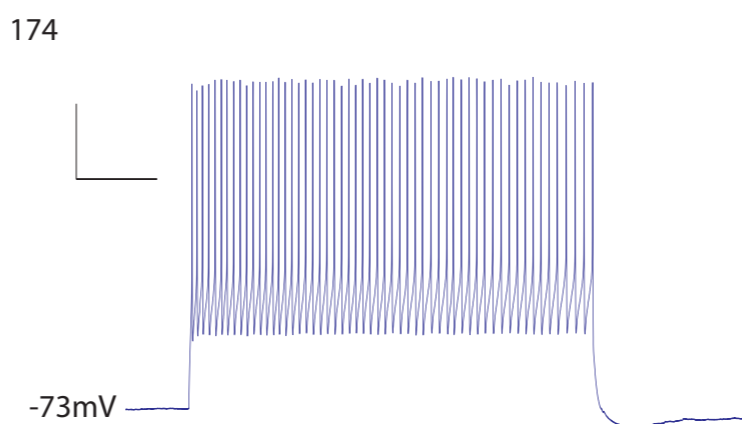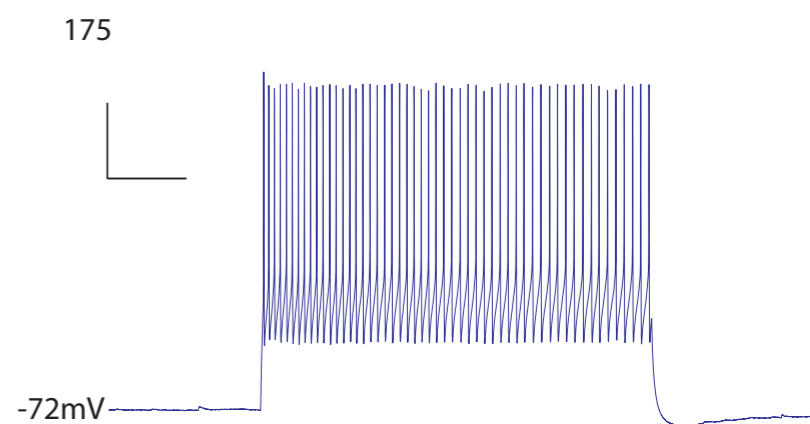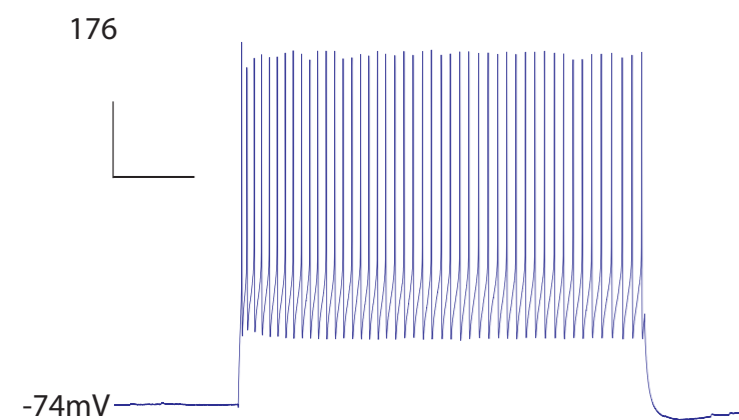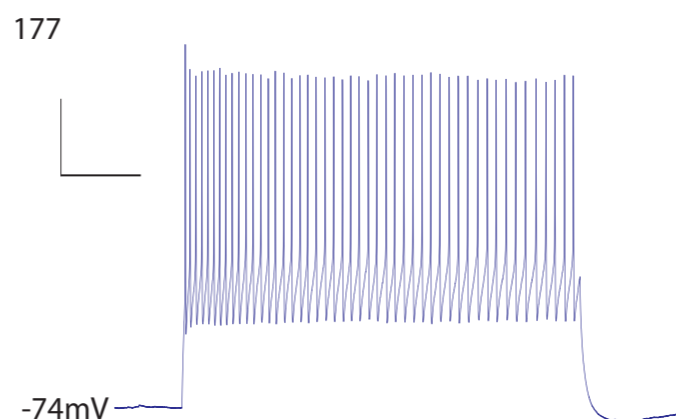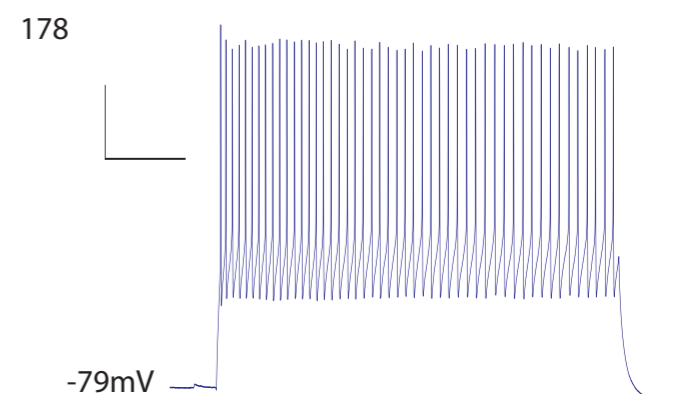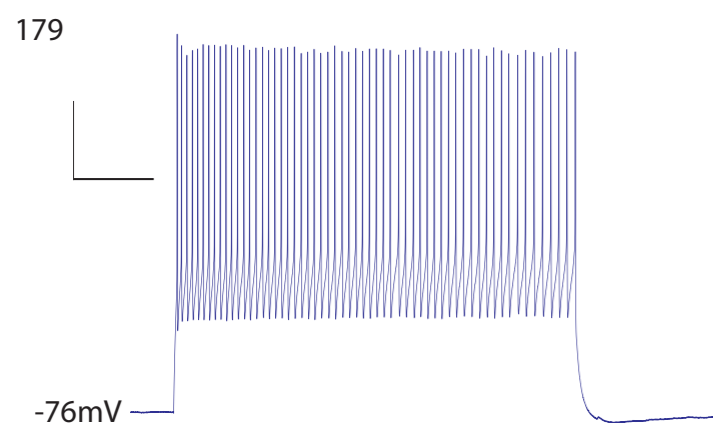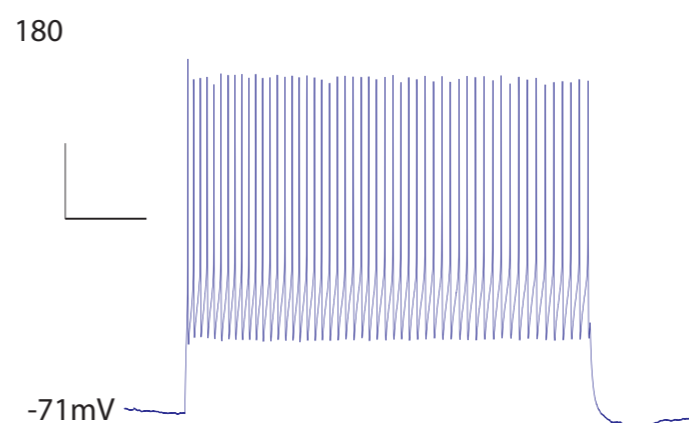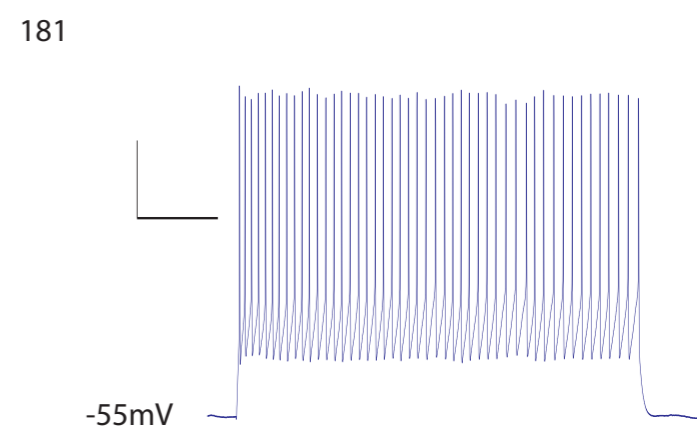

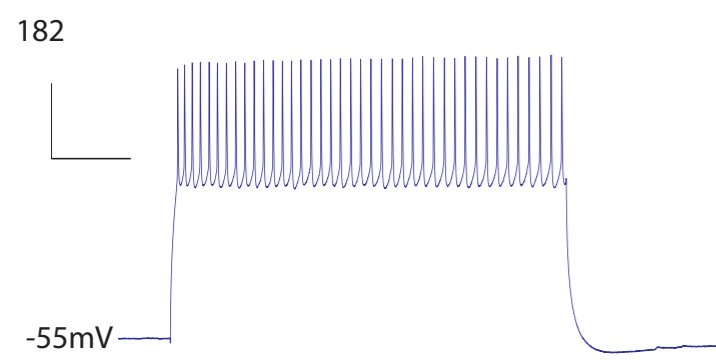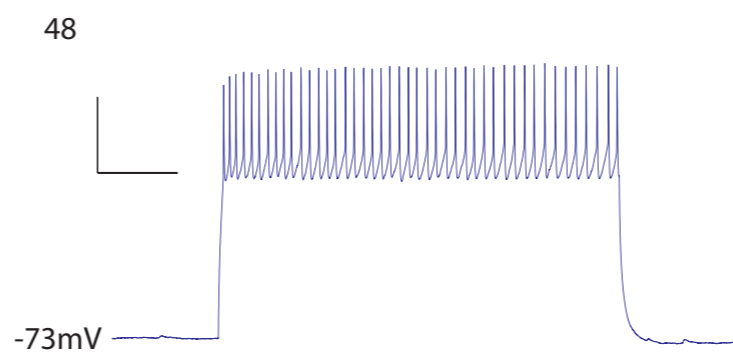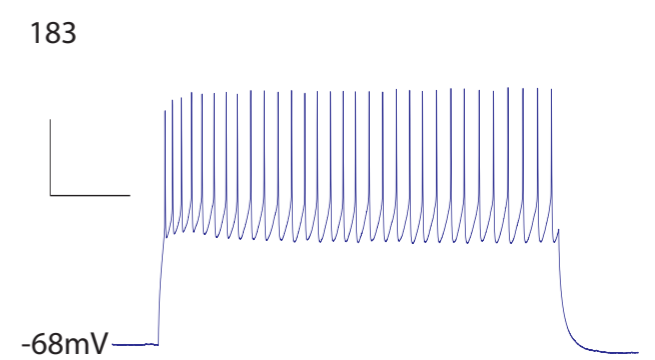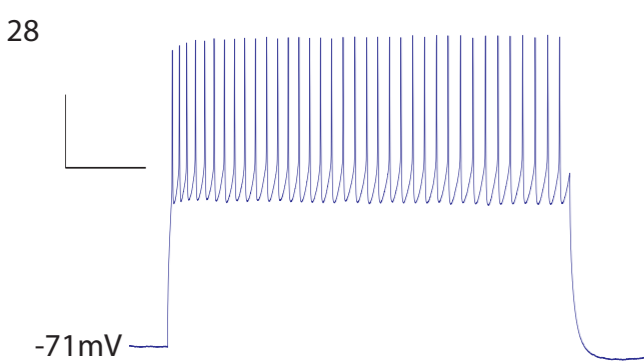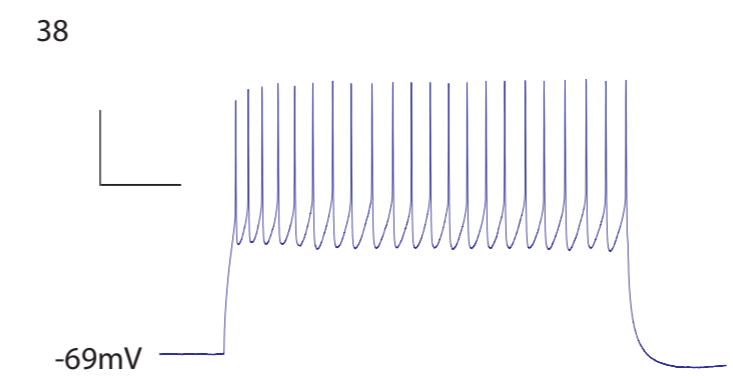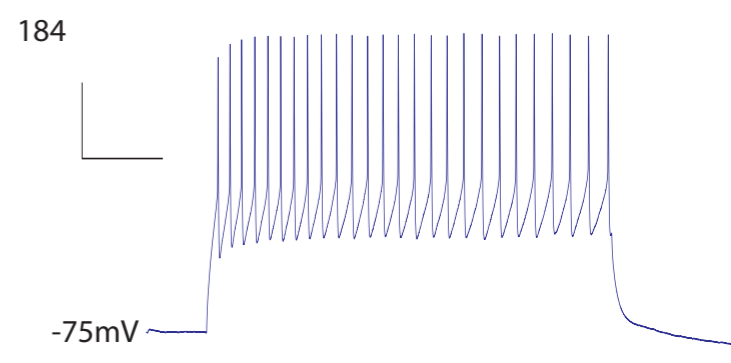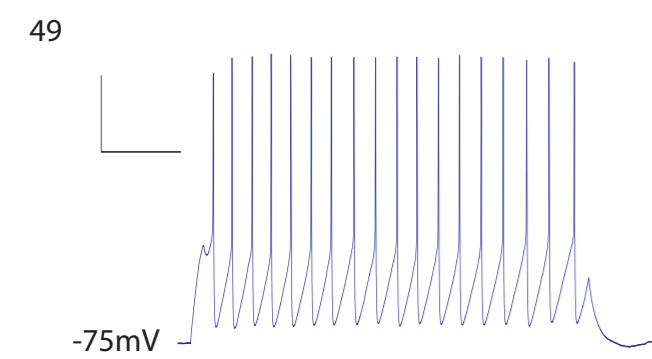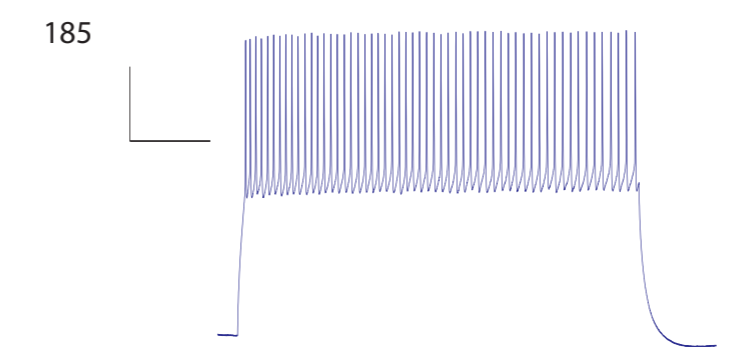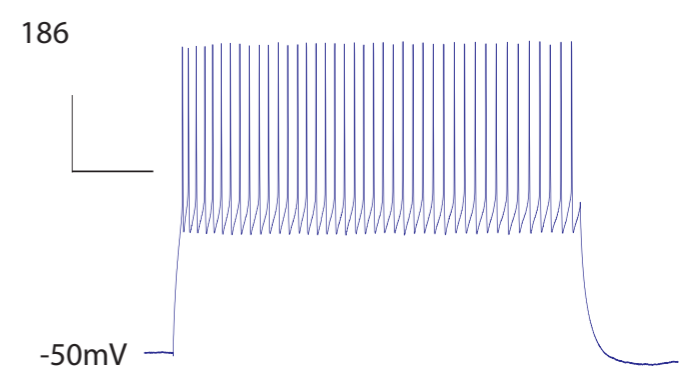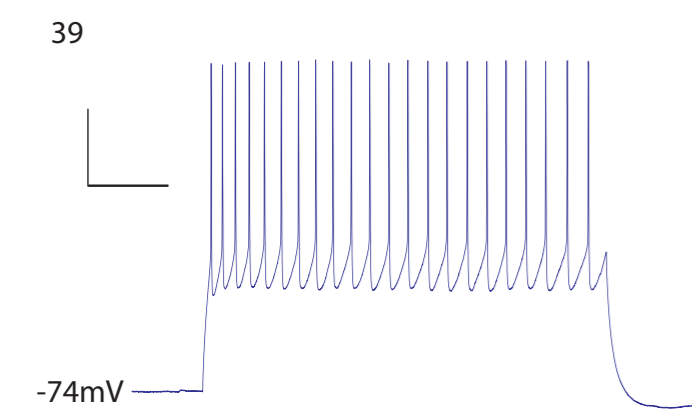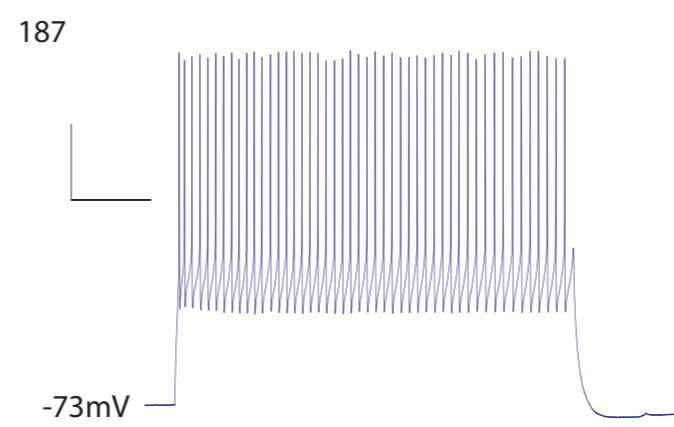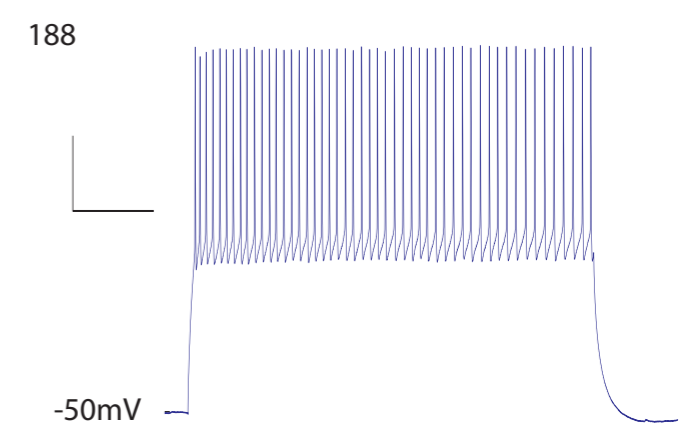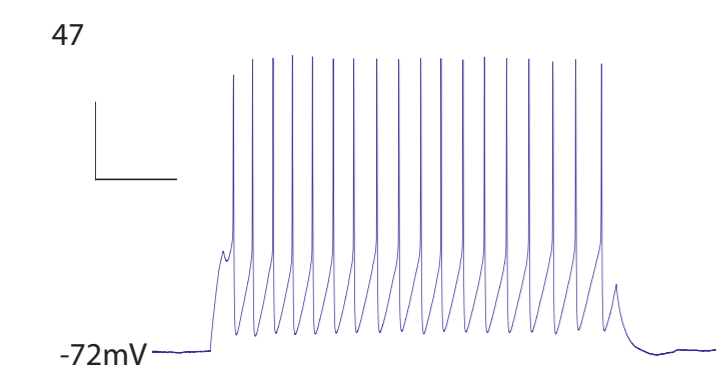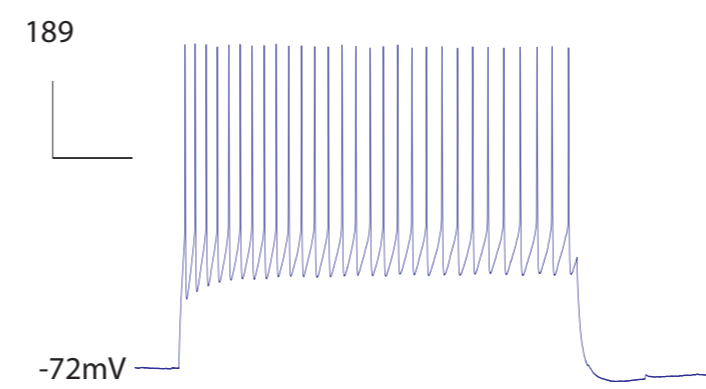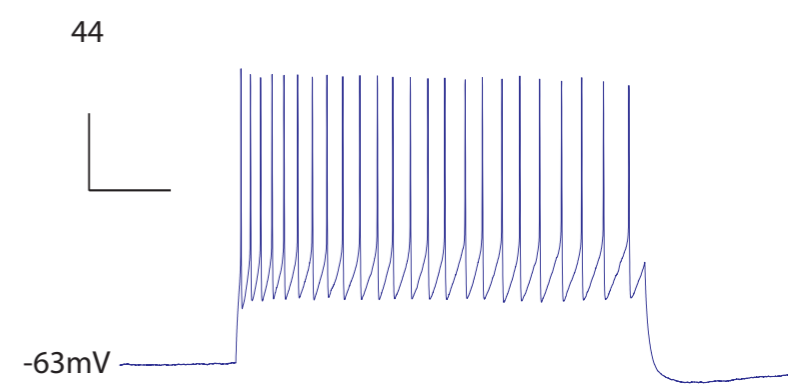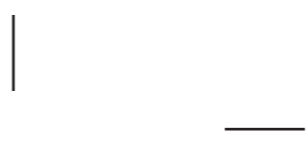

Supplement: Supplementary Figure 1 — Morphological reconstructions of the neurons used. Ordered according to cell type (ChC, BC, MC, and non-MC). [file Presentation1.PDF]

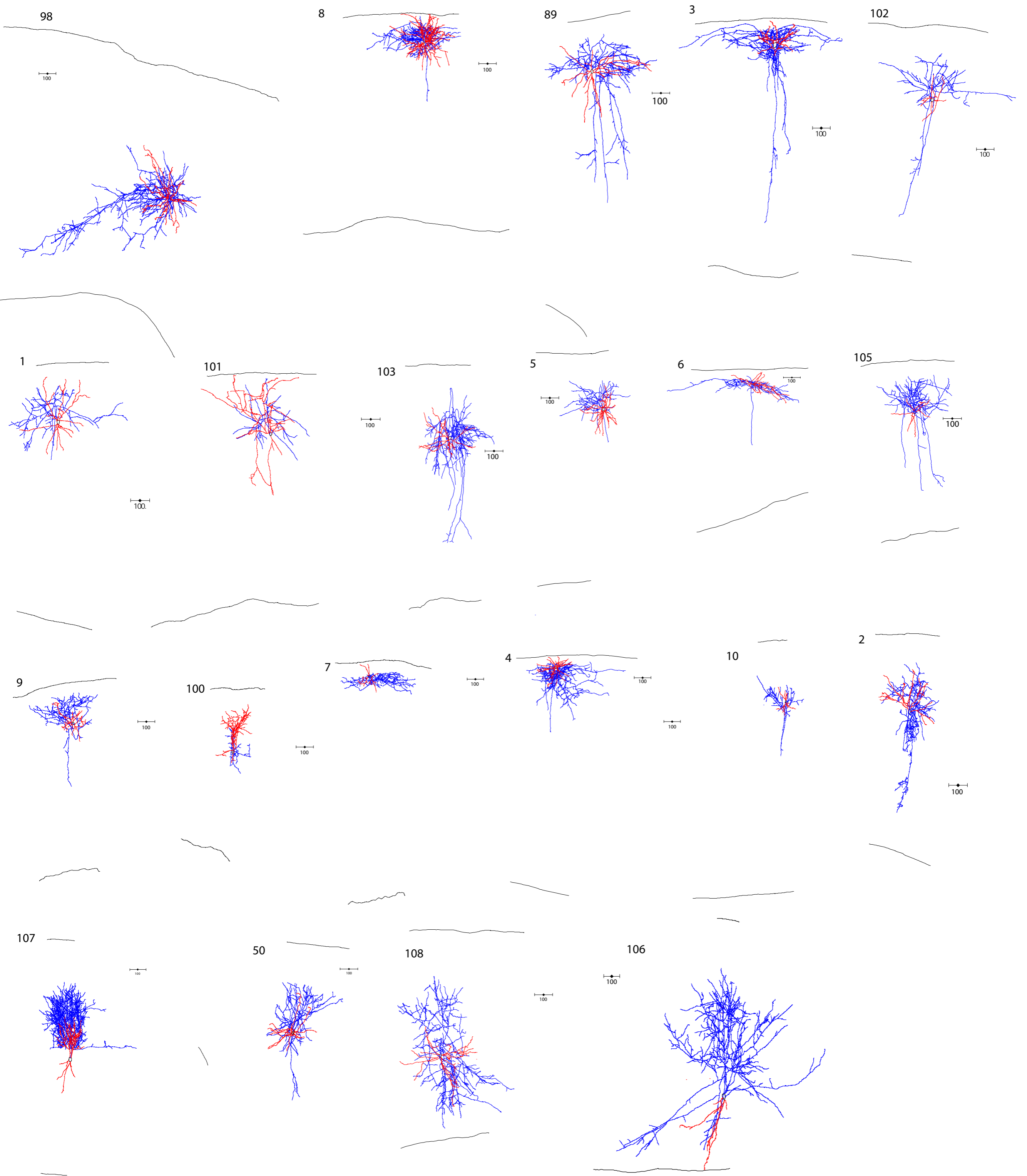

90

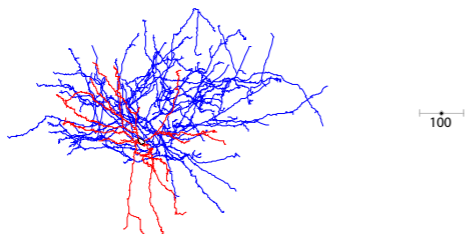

91

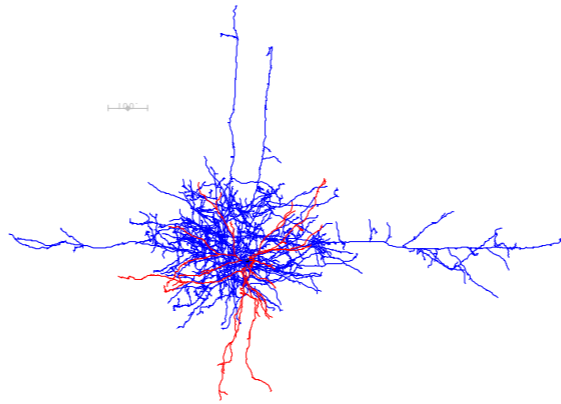

92

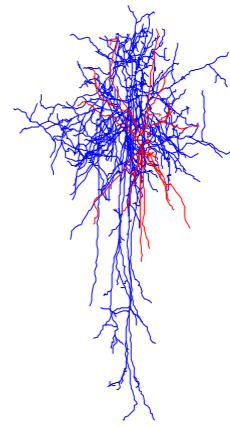

93

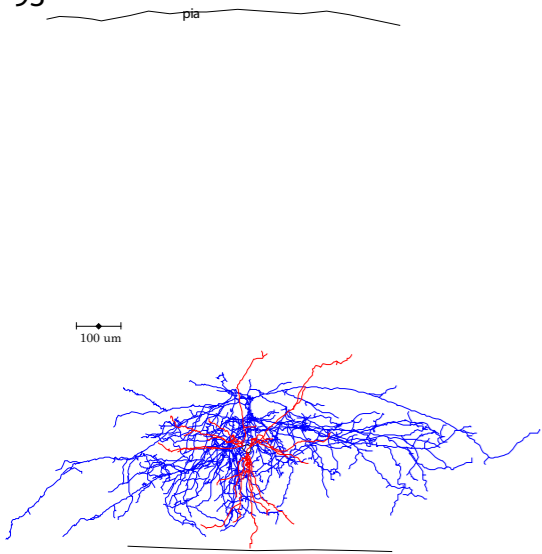

94

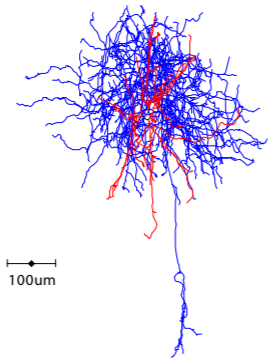

95

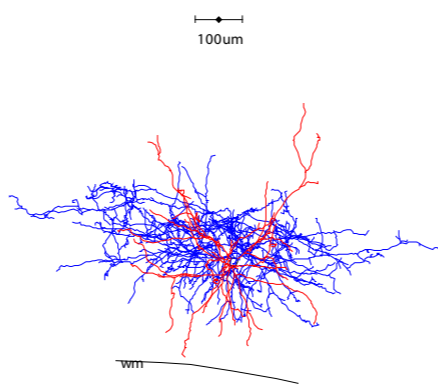

96

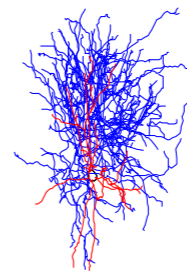

97

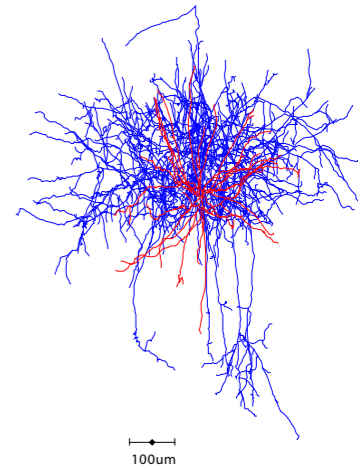

99

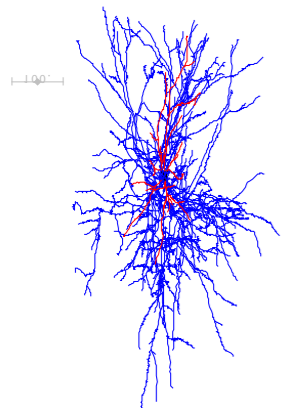

104

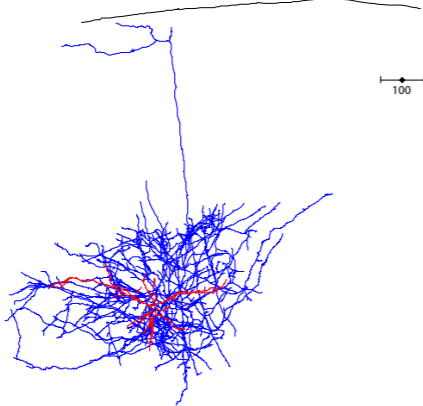

030223-2-PV

27

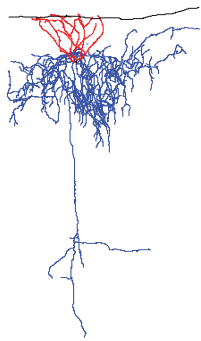

28

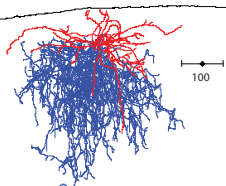

29

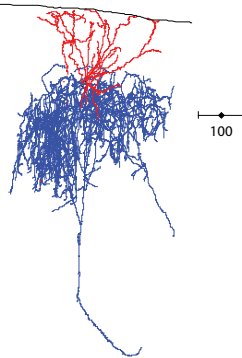

30

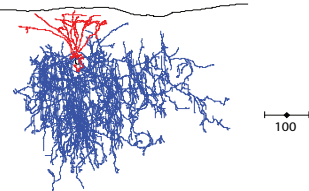

31

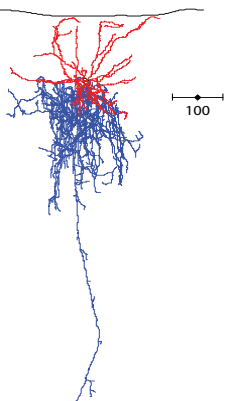

32

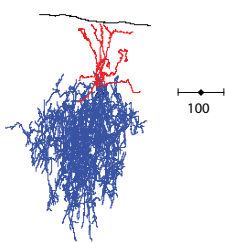

33

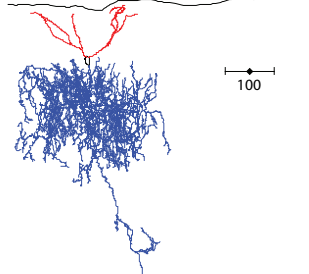

34

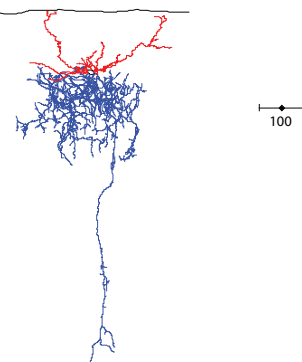

35

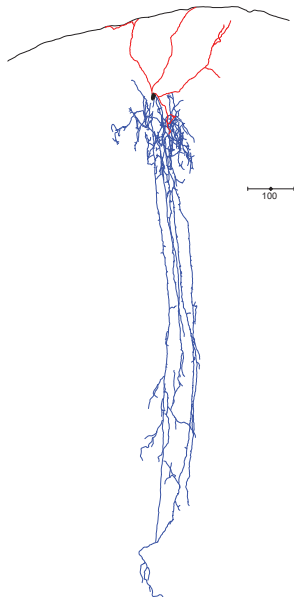

36

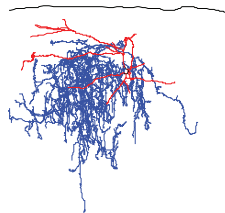

37

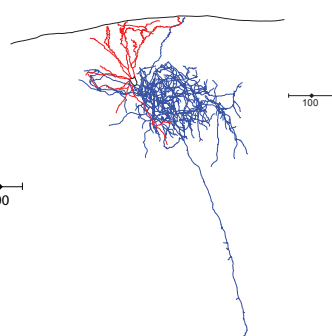

38

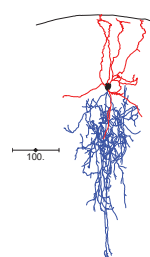

39

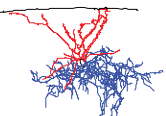

100

40

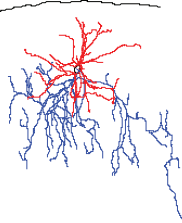

100

41

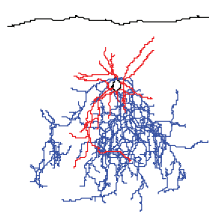

100

42

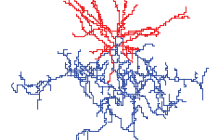

100

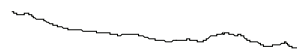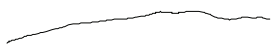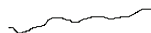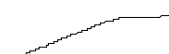

43

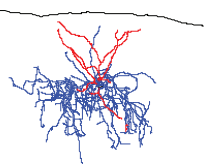

100

44

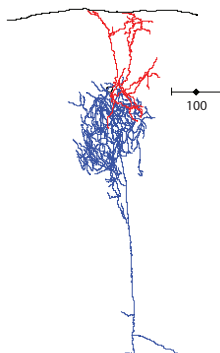

100

45

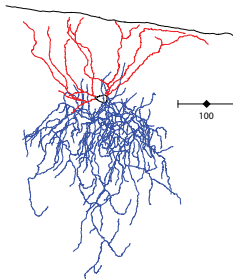

100

46

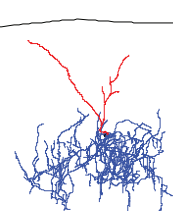

100

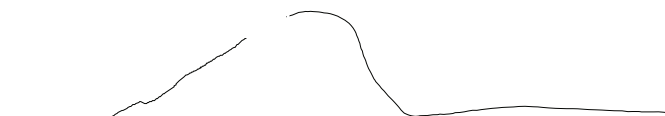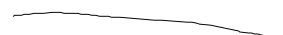

47

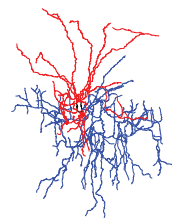

100

48

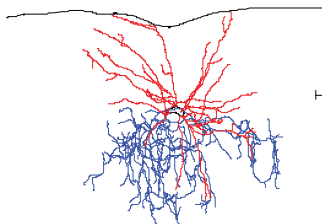

100

49

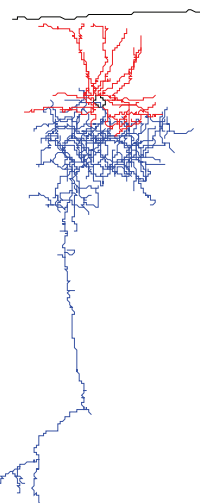

100

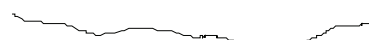

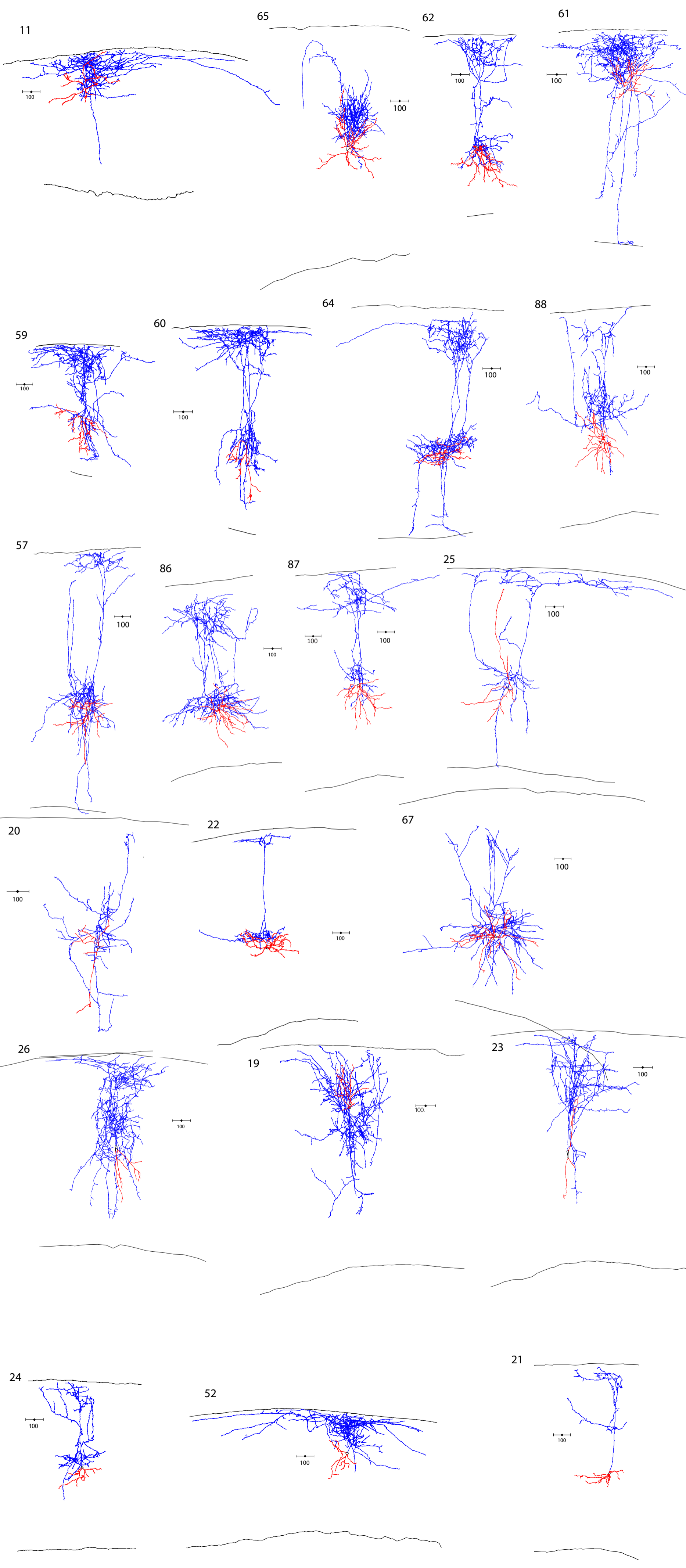

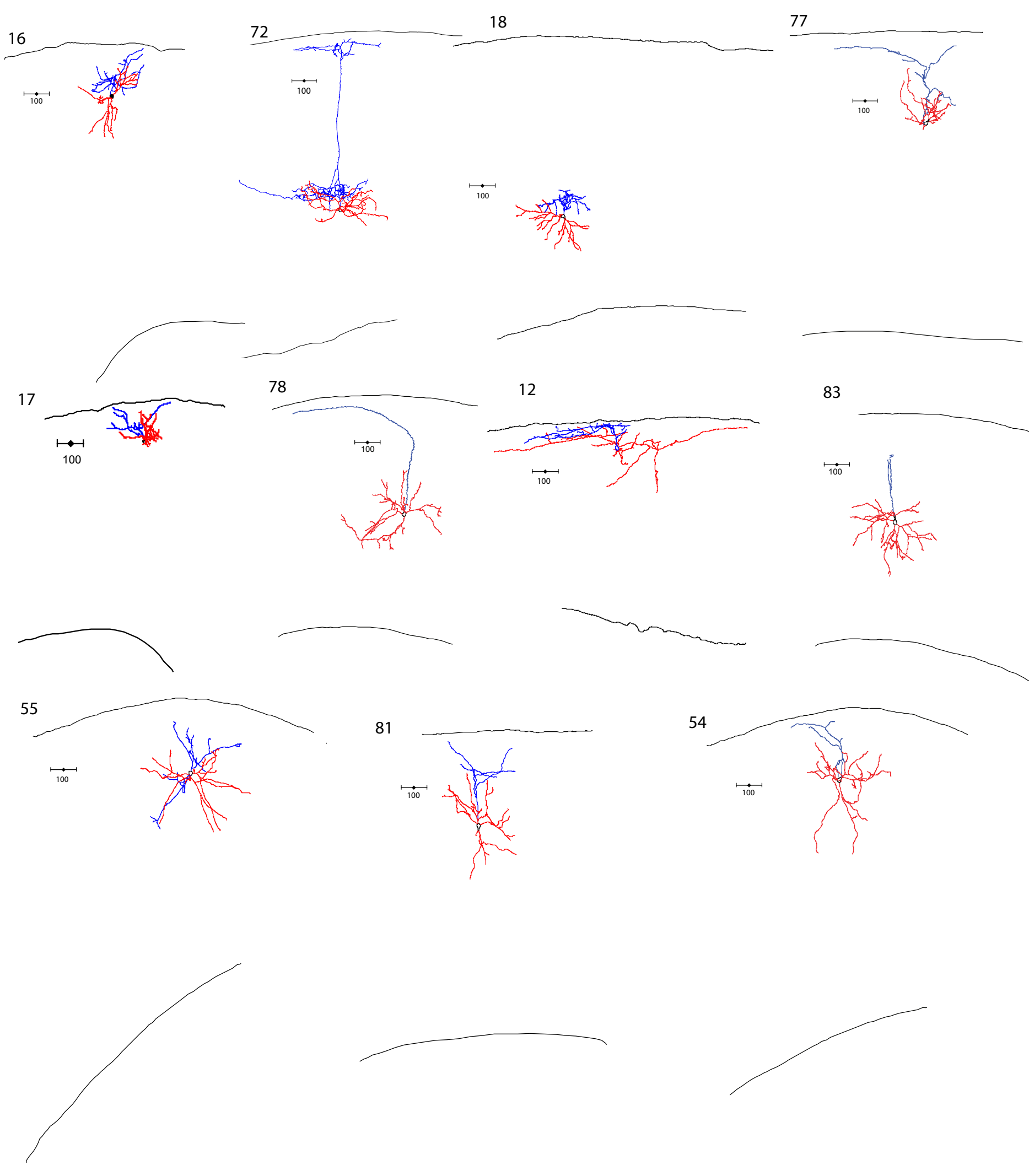

14

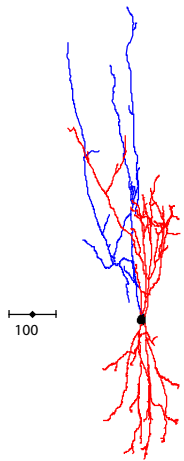

56

100

79

100

15

100

80

100

74

100

13

100

63

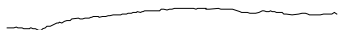

66

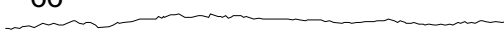

68

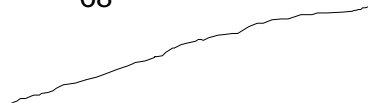

100

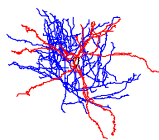

100

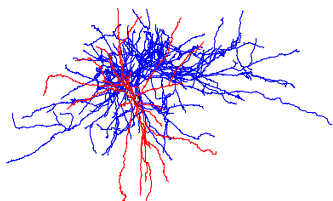

100

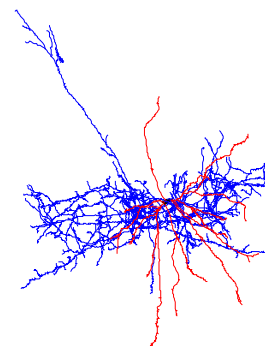

69

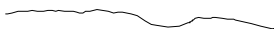

70

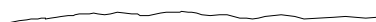

71

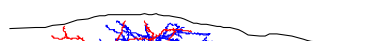

100

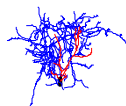

100

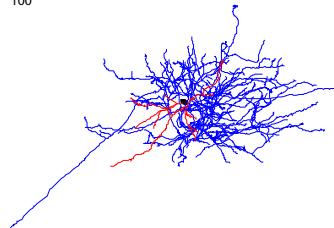

100

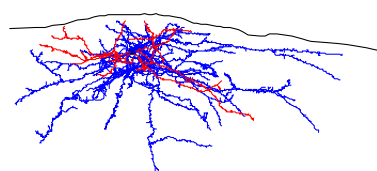

82

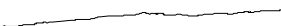

84

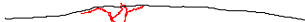

85

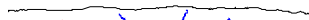

58

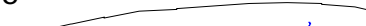

100

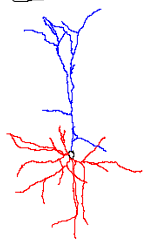

100

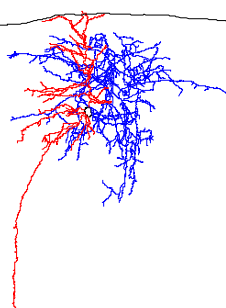

100

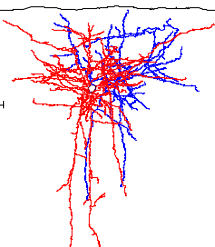

100

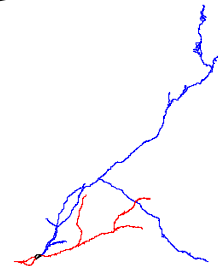

Supplement: Supplementary Figure 2 — Electrophysiological recordings of the neurons used. Ordered according to cell type (ChC, BC, MC, and non-MC). [file Presentation2.PDF]
